# 8-Amino-6-Methoxyquinoline—Tetrazole Hybrids: Impact of Linkers on Antiplasmodial Activity

**DOI:** 10.3390/molecules26185530

**Published:** 2021-09-12

**Authors:** Patrick Hochegger, Johanna Dolensky, Werner Seebacher, Robert Saf, Marcel Kaiser, Pascal Mäser, Robert Weis

**Affiliations:** 1 Institute of Pharmaceutical Sciences, Pharmaceutical Chemistry, University of Graz, Schubertstraße 1, A-8010 Graz, Austria; johanna.faist@uni-graz.at (J.D.); we.seebacher@uni-graz.at (W.S.); robert.weis@uni-graz.at (R.W.); 2 Institute for Chemistry and Technology of Materials (ICTM), Graz University of Technology, Stremayrgasse 9, A-8010 Graz, Austria; robert.saf@tugraz.at; 3 Swiss Tropical and Public Health Institute, Socinstraße 57, CH-4002 Basel, Switzerland; marcel.kaiser@swisstph.ch (M.K.); pascal.maeser@swisstph.ch (P.M.)

**Keywords:** antimalarial, tetrazole derivatives, *Plasmodium falciparum*, 8-aminoquinolines

## Abstract

A new series of compounds was prepared from 6-methoxyquinolin-8-amine or its *N*-(2-aminoethyl) analogue via Ugi-azide reaction. Their linkers between the quinoline and the *tert*-butyltetrazole moieties differ in chain length, basicity and substitution. Compounds were tested for their antiplasmodial activity against *Plasmodium falciparum* NF54 as well as their cytotoxicity against L-6-cells. The activity and the cytotoxicity were strongly influenced by the linker and its substitution. The most active compounds showed good activity and promising selectivity.

## 1. Introduction

Malaria, a vector-borne parasitic disease, is still one of the most dangerous infectious diseases worldwide. With more than 200 million cases in 2019 and 400.000 deaths malaria is a huge burden for mostly sub-Saharan African, South American, Southeast Asian countries [1]. Mostly children under the age of five are prone to a critical and fatal course of disease. Malaria is caused by one of five human pathogens of the genus *Plasmodium* with *Plasmodium falciparum* and *Plasmodium vivax* being responsible for most of the infections. *P. falciparum* is responsible for a majority of fatal outcomes and is therefore considered the deadliest parasite of these five pathogens [2]. Especially *P. falciparum*’s rapid development of resistances against established drugs is of concern. Even against artemisinin derivatives, which are usually combined with other drugs in the first-line treatment of malaria, first resistances are emerging [3,4]. Because of the threat of potentially untreatable malaria infections new active compounds are urgently needed.

The discovery of chloroquine in the 1960s was an essential early turning point to improve malaria treatment. Unfortunately, first resistances were discovered fairly soon. However, chloroquine with its quinoline moiety is still an attractive pharmcophore, possessing limited toxicity, simple cost-effective synthesis and high clinical efficacy [5,6,7]. A promising strategy for developing new antimalarials and circumventing resistances is quinoline hybridization [8,9]

Pandey et al. published a series of compounds where a 4-aminochinoline moiety was linked with a tetrazole ring. In this case an aminophenyl motive functioned as a linker. These compounds possessed antimalarial activities in the submicromolar range. Especially the tetrazole ring was supposed to be important for complexing heme [10]. Recently we published a series of 7-chloroquinoline derivatives that were linked with a tetrazole ring as well as different lipophilic aliphatic and aromatic side chains. A piperidine ring was used as a linker instead of the aminophenyl moiety. These compounds showed distinct antimalarial activities [11].

**Figure 1:**
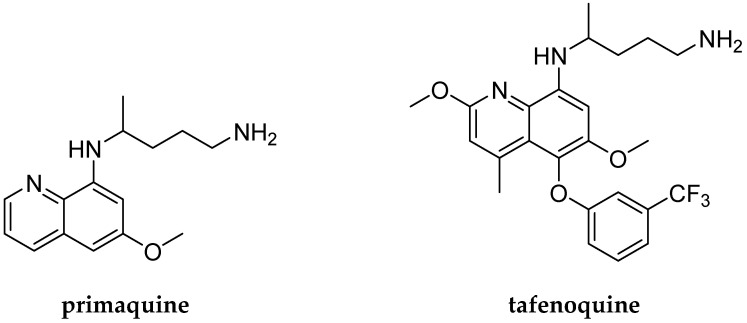
Structures of primaquine and tafenoquine.

The focus of the current work was the synthesis of novel active compounds by using a hybridization approach. The 8-amino-6-methoxyquinoline pharmacophore is part of antimalarials like primaquine and tafenoquine and was therefore used as a central element of the new derivatives. (Figure 1) It was linked to a tetrazole ring via different linkers. Furthermore, lipophilic side chains were varied. All new compounds were characterized and tested in vitro for their activities against *P. falciparum*. The results were compared to those of formerly prepared analogues and of drugs in use.

## 2. Results and Discussion

### 2.1. Chemistry

As precursor for all final compounds served 6-methoxyquinolin-8-amine **2**, which was synthesized in a two-step reaction from 4-methoxy-2-nitroaniline and glycerol in a so called Skraup reaction [12]. This reaction resulted in 6-methoxy-8-nitroquinoline **1**, which was further reduced by SnCl_2_ to 6-methoxyquinolin-8-amine **2** [13].

Compounds **13**–**22** were obtained by an Ugi-azide reaction of amine **2** with *tert*-butyl isocyanide, trimethylsilyl azide and various aldehydes to investigate the influence of different side chains adjacent to the newly formed tetrazole group [14].

In case of compounds **7**–**12** an additional ethyl linker was integrated into the molecule to examine how a greater distance between the 8-amino group of the quinoline moiety and the tetrazole ring impacts the activity. A straightforward nucleophilic substitution reaction of compound **2** with 2-chloroethyl-1-amine hydrochloride to amine **6** did not give any product. Therefore, we looked for a different approach and the linker was synthesized via multiple steps. At first compound **2** reacted with chloroacetyl chloride to an amide **3** [15,16]. The terminal chloro group of the newly formed compound was subsequently substituted by an azide group using sodium azide in DMF as solvent [16]. This azide group of **4** was converted to an amine in a Staudinger reaction by using triphenylphosphine [16]. In a final reaction with LiAlH_4_ the amide group of **5** was reduced yielding amine **6** [17]. Different synthesis methods for compounds **3**, **5** and **6** are known, but without any NMR data given for these substances [18,19,20,21].

**Scheme 1:**
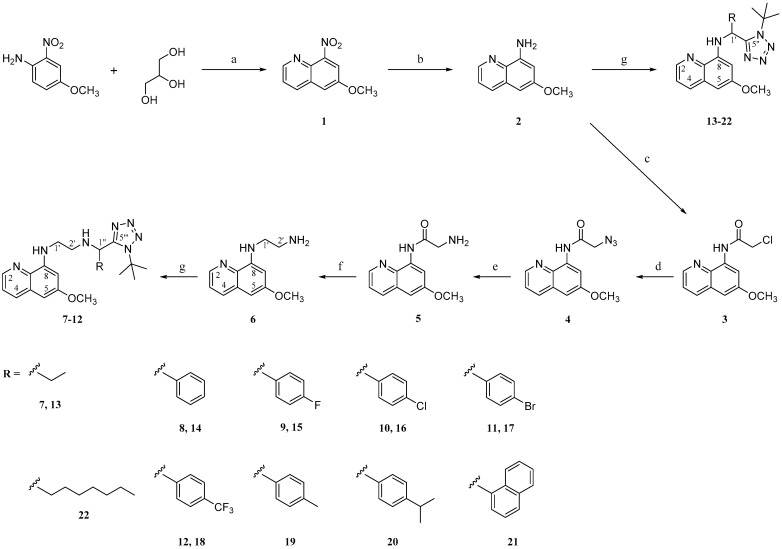
Preparation of compounds **1**–**22**, Reagents and conditions: (a) FeSO_4_ × 7 H_2_O, methanesulfonic acid, 130 °C, 6 h; (b) SnCl_2_ × 2 H_2_O, EtOH, rt, 48 h; (c) chloroacetyl chloride, triethylamine, CH_2_Cl_2_, rt, 20 h; (d) NaN_3_, DMF, rt, 48 h; (e) triphenylphosphine, THF, H2O, reflux, 20 h; (f) LiAlH_4_, diethyl ether, rt, 20 h; (g) aldehyde, trimethylsilyl azide, *tert*-butyl isocyanide, MeOH, rt, 20–120 h.

Afterwards compound **6** was converted to compounds **7**–**12** via Ugi-azide reactions. For this reaction the aldehyde component was varied to obtain compounds with different aliphatic and aromatic side chains close to the tetrazole moiety (Scheme 1) [14].

The structures of all newly synthesized compounds were clarified by one- and two-dimensional NMR spectroscopy. Products of the Ugi-azide reaction showed characteristic signals in the NMR spectra of compounds **7**–**22**. Their *tert*-butyl group gave an intense proton resonance at low frequencies as well as carbon resonances at about 30 and 61.5 ppm. A longrange coupling was observed from their new methine proton to the tetrazole carbon which gave resonances at 155–157 ppm depending on substitution of the methine carbon atom. ^1^H- and ^13^C-NMR-spectra are given in the Supplementary Materials.

### 2.2. Antiplasmodial Activity and Cytotoxicity

All compounds were tested for their antiplasmodial activity against the chloroquine-sensitive strain NF54 of *P. falciparum*. Further on their cytotoxicity was determined using skeletal myoblasts (L-6 cells). As standards served chloroquine and podophyllotoxin (Table 1).

The prepared compounds showed heterogeneous activities against *P. falciparum* NF54. Compounds **7**–**12** with a basic (methylamino)ethyl linker were usually way less active (*Pf*NF54 IC_50_ = 15.98–2.51 µM) than compounds **15**–**22** with a methyl linker (*Pf*NF54 IC_50_ = 2.68–0.324 µM) with the exception of compounds **13** and **14** (*Pf*NF54 IC_50_ = 23.60–5.12 µM).

In the case of compounds **7**–**12** the least active compound was **7** (*Pf*NF54 IC_50_ = 15.98 µM) with an ethyl side chain. A more lipophilic phenyl ring as side chain improved antiplasmodial activity in general, which is apparent in compounds **8**–**12** (*Pf*NF54 IC_50_ = 7.05–2.51 µM). In this series the most active compounds were **11** and **12** (*Pf*NF54 IC_50_ = 2.92–2.51 µM) with a 4-bromophenyl and a 2-(trifluoromethyl)phenyl side chain, respectively. Replacement of the bromine atom by a hydrogen, a fluorine or a chlorine atom led to less active compounds **8**–**10** (*Pf*NF54 IC_50_ = 7.05–5.34 µM).

Compounds **13**–**22** with the methyl linker were generally more active. The least active compound **13** (*Pf*NF54 IC_50_ = 23.60 µM) was again the one with the ethyl side chain. Its more lipophilic phenyl analogue **14** (*Pf*NF54 IC_50_ = 5.12 µM) showed improved, but still weak activity. Substitution in ring position 4 of the phenyl ring with a fluorine atom, a methyl or an isopropyl group as well as its replacement with a 1-naphthyl moiety further increased the antiplasmodial activity (**15** and **19**–**21** *Pf*NF54 IC_50_ = 2.68–1.04 µM). Compounds with bulkier electron withdrawing substituents like **16**–**18** (*Pf*NF54 IC_50_ = 0.743–0.464 µM) were even more active. The most active compound **22** (*Pf*NF54 IC_50_ = 0.324 µM) possesses a heptyl side chain. This indicates a positive impact of highly lipophilic and voluminous side chains. However, an additional stereochemical effect could be relevant, because the 1-naphthyl analogue **21** (*Pf*NF54 IC_50_ = 1.26 µM) was significantly less active. Compounds **15** and **20** (*Pf*NF54 IC_50_ = 2.68–2.00 µM) showed low cytotoxicity (L-6 cells IC_50_ = 119.4–124.0 µM) and good selectivity (S.I. = 46.27–59.7). The most active compounds **16**–**19** and **21**–**22** (*Pf*NF54 IC_50_ = 1.26–0.324 µM) also showed low cytotoxicity (L-6 cells IC_50_ = 54.73–248.5 µM) resulting in promising selectivity (S.I. = 137.6–318.3).

## 3. Materials and Methods

### 3.1. Instrumentation and Chemicals

Materials: Solvents and reagents were used without further purification. Dry solvents were either purchased in sealed bottles or were dried over molecular sieves or dried with sodium. Column chromatography (CC): silica gel 60 (Merck 70–230 mesh, pore diameter 60 Å), aluminium oxide (pH: 9.5, Fluka). Thin-layer chromatography (TLC): TLC plates silica gel 60 F254 (Merck), aluminium oxide 60 F254 (neutral, Merck). Melting points were obtained on an Electrothermal IA 9200 melting point apparatus. IR spectra: Bruker Alpha Platinum ATR FTIR spectrometer (KBr discs); frequencies are reported in cm^−1^. The structures of all newly synthesized compounds were determined by one- and two-dimensional NMR spectroscopy. NMR spectra: Varian UnityInova 400 (298 K) 5 mm tubes, TMS as internal standard. Shifts in ^1^H NMR (400 MHz) and ^13^C NMR (100 MHz) spectra are reported in ppm; ^1^H- and ^13^C-resonances were assigned using ^1^H,^1^H- and ^1^H,^13^C-correlation spectra and are numbered as given in Scheme 1. Signal multiplicities are abbreviated as follows: br, broad; d, doublet; dd, doublet of doublets; ddd, doublet of doublet of doublets; dt, doublet of triplets; m, multiplet; s, singlet; t, triplet; td, triplet of doublets; q, quartet. HRMS: Micromass Tofspec 3E spectrometer (MALDI) and GCT-Premier, Waters (EI, 70eV). ^1^H-NMR and ^13^C-NMR spectra of new compounds are available in Supplementary Materials (Figures S1–S20).

### 3.2. Syntheses

Compounds 6-methoxy-8-nitroquinoline **1**, and 6-methoxyquinoline-8-amine **2** were synthesized according to an already published protocol and their NMR data were in accordance with literature data [12,13].

*2-Chloro-N-(6-methoxyquinolin-8-yl)acetamide* (**3**): 6-Methoxyquinolin-8-amine **2** (0.523 g (3.00 mmol)) was dissolved in in dry CH_2_Cl_2_ (15 mL) and cooled to 0 °C with an ice bath. Triethylamine (2.079 mL (15.00 mmol)) was added and the mixture was stirred for 10 min. Chloroacetyl chloride (0.477 mL (6.00 mmol)) in dry CH_2_Cl_2_ (15 mL) was added dropwise via a dropping funnel. The ice bath was removed and the reaction mixture stirred at 25 °C for 20 h. Then, the reaction was quenched with 2*N* NaOH at 0 °C and the mixture was basified to a pH of 10–11. The aqueous and organic phases were separated and the aqueous phase was extracted with CH_2_Cl_2_. The combined organic phases were washed with 0.1% aqueous NaHCO_3_ and dried over anhydrous sodium sulfate and filtered. The solvent was removed in vacuo and the obtained raw product purified by column chromatography (silica gel, diethyl ether) to yield compound **3** as off-white amorphous solid (0.700 g (93%)). ^1^H NMR (CDCl_3_, 400 MHz) *δ* = 3.72 (s, 3H, OCH_3_), 4.15 (s, 2H, CH_2_Cl), 6.58 (d, *J* = 2.6 Hz, 1H, 5-H), 7.19 (dd, *J* = 8.2, 4.2 Hz, 1H, 3-H), 7.80 (dd, *J* = 8.2, 1.6 Hz, 1H, 4-H), 8.25 (d, *J* = 2.6 Hz, 1H, 7-H), 8.46 (dd, *J* = 4.1, 1.6 Hz, 1H, 2-H), 10.60 (s, 1H, NH); ^13^C NMR (CDCl_3_, 100 MHz) *δ* = 43.10 (CH_2_Cl), 55.26 (OCH_3_), 100.05 (C-5), 108.91 (C-7), 121.90 (C-3), 128.51 (C-4a), 133.97 (C-8), 134.55 (C-4), 134.78 (C-8a), 145.69 (C-2), 157.75 (C-6), 164.03 (CO).

*2-Azido-N-(6-methoxyquinolin-8-yl)acetamide* (**4**): Compound **3** (0.702 g (2.80 mmol)) was dissolved in dry DMF (30 mL) and cooled to 0 °C with an ice bath. Sodium azide (0.364 g (5.60 mmol)) was added in small portions and after that the ice bath was removed and the reaction mixture stirred at 25 °C for 48 h. Then, the reaction was quenched with 0.1% aqueous NaHCO_3_ (50 mL). The aqueous phase was extracted with ethyl acetate. The combined organic phases were washed with 0.1% aqueous NaHCO_3_ and dried over anhydrous sodium sulfate and filtered. The solvent was removed in vacuo and the obtained raw product purified by column chromatography (silica gel, ethyl acetate/ethanol 6:1) to yield compound **4** as off-white amorphous solid (0.634 g (88%)). ^1^H NMR (CDCl_3_, 400 MHz) *δ* = 3.85 (s, 3H, OCH_3_), 4.21 (s, 2H, CH_2_N_3_), 6.71 (d, *J* = 2.6 Hz, 1H, 5-H), 7.33 (dd, *J* = 8.2, 4.3 Hz, 1H, 3-H), 7.93 (dd, *J* = 8.2, 1.6 Hz, 1H, 4-H), 8.40 (d, *J* = 2.6 Hz, 1H, 7-H), 8.60 (dd, *J* = 4.3, 1.6 Hz, 1H, 2-H), 10.38 (br s, 1H, NH); ^13^C NMR (CDCl_3_, 100 MHz) *δ* = 52.93 (CH_2_N_3_), 55.10 (OCH_3_), 99.80 (C-5), 108.88 (C-7), 121.74 (C-3), 128.41 (C-4a), 133.84 (C-8), 134.45 (C-4), 134.53 (C-8a), 145.49 (C-2), 157.65 (C-6), 164.70 (CO).

*2-Amino-N-(6-methoxyquinolin-8-yl)acetamide* (**5**): Compound **4** (0.630 g (2.45 mmol)) was dissolved in THF/H_2_O 6:1 (40 mL) at room temperature. Triphenylphosphine (1.26 g (4.90 mmol)) was added in small portions. Followed by that the brown reaction mixture was refluxed for 20 h. The mixture was cooled to room temperature and the solvent removed in vacuo. The residue was adsorbed on a column with silica gel and excess triphenylphosphine and triphenylphospine oxide were removed by elution with diethyl ether. Elution of the product with CH_2_Cl_2_/*Me*OH 1:1 afforded a raw product which was further purified by column chromatography (silica gel, ethyl acetate/ethanol 6:1) to yield compound **5** as orange amorphous solid (0.504 g (89%)). ^1^H NMR (CDCl_3_, 400 MHz) *δ* = 1.75 (br s, 2H, NH_2_), 3.65 (s, 2H, CH_2_), 3.93 (s, 3H, OCH_3_), 6.81 (d, *J* = 2.8 Hz, 1H, 5-H), 7.39 (dd, *J* = 8.3, 4.2 Hz, 1H, 3-H), 8.03 (d, *J* = 8.3, 1.5 Hz, 1H, 4-H), 8.57 (d, *J* = 2.8 Hz, 1H, 7-H), 8.70 (dd, *J* = 4.2, 1.5 Hz, 1H, 2-H), 11.25 (br s, 1H, NH); ^13^C NMR (CDCl_3_, 100 MHz) *δ* = 46.09 (CH_2_), 55.45 (OCH_3_), 99.91 (C-5), 108.92 (C-7), 121.95 (C-3), 129.03 (C-4a), 134.92 (C-4), 135.11 (C-8), 135.53 (C-8a), 145.94 (C-2), 158.37 (C-6), 171.55 (CO).

*N^1^-(6-Methoxyquinolin-8-yl)ethan-1,2-diamine* (**6**): Compound **5** (0.509 g (2.20 mmol)) was dissolved in dry diethyl ether (30 mL) and cooled to 0 °C with an ice bath. Pulverized LiAlH_4_ (0.167 g (4.40 mmol)) was added slowly in small portions and the reaction mixture was stirred for 30 min at 0 °C. Then the mixture was refluxed for 20 h. The suspension was cooled to 0 °C and quenched with 2*N* NaOH and was basified to a pH of 10–11. The aqueous and organic phases were separated and the aqueous phase was extracted with diethyl ether. The combined organic phases were dried over anhydrous sodium sulfate and filtered. The solvent was removed in vacuo and the obtained raw product purified by column chromatography (silica gel, CH_2_Cl_2_/*Me*OH 5:1) to yield compound **6** as off-white amorphous solid (0.277 g (58%)). ^1^H NMR (CDCl_3_, 400 MHz) *δ* = 1.40 (br s, 2H, NH_2_), 3.07 (t, *J* = 6.0 Hz, 2H, 2′-H), 3.37 (q, *J* = 6.0 Hz, 2H, 1′-H), 3.89 (s, 3H, OCH_3_), 6.31–6.38 (m, 1H, NH), 6.34 (d, *J* = 2.5 Hz, 1H, 7-H), 6.37 (d, *J* = 2.5 Hz, 1H, 5-H), 7.31 (dd, *J* = 8.3, 4.2 Hz, 1H, 3-H), 7.93 (dd, *J* = 8.3, 1.2 Hz, 1H, 4-H), 8.55 (dd, *J* = 4.2, 1.2 Hz, 1H, 2-H); ^13^C NMR (CDCl_3_, 100 MHz) *δ* = 40.93 (C-2′), 46.18 (C-1′), 55.08 (OCH_3_), 92.10 (C-5), 96.76 (C-7), 121.73 (C-3), 129.64 (C-4a), 134.62 (C-4), 135.29 (C-8a), 144.35 (C-2), 145.78 (C-8), 159.25 (C-6).

The general procedure for the Ugi-azide reaction (**7**–**22**) is as follows: 6-Methoxyquinoline-8-amine **2** (0.75 mmol) or compound **6** (0.75 mmol) were dissolved in dry methanol (5 mL). The corresponding aldehyde (0.75 mmol) was added and the mixture stirred at room temperature for 1 h under an argon atmosphere. Trimethylsilyl azide (0.75 mmol) and *tert*-butyl isocyanide (0.75 mmol) were added dropwise and the reaction mixture was stirred for 20–120 h. After that, the solvent was evaporated in vacuo and the residue was dissolved in CH_2_Cl_2_. The solution was washed several times with 30% aqueous sodium disulfite followed by 0.1% aqueous NaHCO_3_. The organic phase was dried over anhydrous sodium sulfate and filtered. The solvent was removed in vacuo, yielding the raw products **7**–**22**, which were further purified by column chromatography.

*N^1^-(6-Methoxyquinolin-8-yl)-N^2^-(1-(tert-butyl-1H-tetrazol-5-yl)propyl)ethan-1,2-diamine* (**7**): The reaction of compound **6** (163 mg (0.75 mmol)), propanal (44 mg, 54 µL (0.75 mmol)), trimethylsilyl azide (86 mg, 100 µL (0.75 mmol)) and *tert*-butyl isocyanide (62 mg, 85 µL (2.00 mmol)) in dry methanol (5 mL) gave the raw tetrazole which was purified by column chromatography (silica gel, diethyl ether) to yield compound **7** as yellow oil (63 mg (22%)). IR = 2959, 1618, 1579, 1519, 1459, 1425, 1390, 1211, 1152, 1115, 918, 828, 794; ^1^H NMR (CDCl_3_, 400 MHz) *δ* = 1.04 (t, *J* = 7.3 Hz, 3H, 3″-H), 1.72 (s, 9H, (CH_3_)_3_), 1.87–1.97 (m, 3H, 2″-H, NH), 2.83 (dt, *J* = 11.9, 6.0 Hz, 1H, 2′-H), 2.92 (dt, *J* = 11.9, 5.8 Hz, 1H, 2′-H), 3.30–3.37 (m, 2H, 1′-H), 3.88 (s, 3H, OCH_3_), 4.17 (t, J = 6.6 Hz, 1H, 1″-H), 6.28 (d, *J* = 2.5 Hz, 1H, 7-H), 6.36 (d, *J* = 2.5 Hz, 1H, 5-H), 6.44–6.49 (m, 1H, NH), 7.30 (dd, *J* = 8.3, 4.2 Hz, 1H, 3-H), 7.92 (dd, *J* = 8.3, 1.4 Hz, 1H, 4-H), 8.53 (dd, *J* = 4.2, 1.4 Hz, 1H, 2-H); ^13^C NMR (CDCl_3_, 100 MHz) *δ* = 10.81 (C-3″), 28.87 (C-2″), 30.17 ((CH_3_)_3_), 43.21 (C-1′), 45.94 (C-2′), 55.17 (OCH_3_), 55.70 (C-1″), 61.04 (C*Me*_3_), 92.26 (C-5), 96.89 (C-7), 121.82 (C-3), 129.67 (C-4a), 134.61 (C-4), 135.43 (C-8a), 144.47 (C-2), 145.79 (C-8), 157.40 (C-5‴), 159.30 (C-6); HRMS (EI+) calcd for C_20_H_29_N_7_O: 383.2433; found: 383.2441.

*N^1^-(6-Methoxyquinolin-8-yl)-N^2^-((phenyl)(tert-butyl-1H-tetrazol-5-yl)methyl)ethan-1,2-diamine* (**8**): The reaction of compound **6** (163 mg (0.75 mmol)), benzaldehyde (80 mg, 76 µL (0.75 mmol)), trimethylsilyl azide (86 mg, 100 µL (0.75 mmol)) and *tert*-butyl isocyanide (62 mg, 85 µL (2.00 mmol)) in dry methanol (5 mL) gave the raw tetrazole which was purified by column chromatography (silica gel, diethyl ether) to yield compound **8** as orange oil (87 mg (27%)). IR = 2937, 1578, 1520, 1454, 1423, 1388, 1213, 1154, 1108, 1028, 823, 792; ^1^H NMR (CDCl_3_, 400 MHz) *δ* = 1.57 (s, 9H, (CH_3_)_3_), 2.40 (br, 1H, NH), 2.90 (dt, *J* = 12.0, 6.0 Hz, 1H, 2′-H), 3.02 (dt, *J* = 12.0, 6.0 Hz, 1H, 2′-H), 3.37–3.42 (m, 2H, 1′-H), 3.88 (s, 3H, OCH_3_), 5.38 (s, 1H, 1″-H), 6.30 (d, *J* = 2.5 Hz, 1H, 7-H), 6.37 (d, *J* = 2.5 Hz, 1H, 5-H), 6.50 (br, 1H, NH), 7.27–7.35 (m, 6H, 3-H, aromatic H), 7.93 (dd, *J* = 8.2, 1.5 Hz, 1H, 4-H), 8.55 (dd, *J* = 4.1, 1.5 Hz, 1H, 2-H); ^13^C NMR (CDCl_3_, 100 MHz) *δ* = 29.90 ((CH_3_)_3_), 43.12 (C-1′), 46.34 (C-2′), 55.20 (OCH_3_), 58.78 (C-1″), 61.33 (C*Me*_3_), 92.32 (C-5), 96.95 (C-7), 121.86 (C-3), 128.11, 128.35, 128.91 (aromatic C), 129.70 (C-4a), 134.64 (C-4), 135.43 (C-8a), 138.64 (aromatic C_q_), 144.50 (C-2), 145.73 (C-8), 155.51 (C-5‴), 159.32 (C-6); HRMS (EI+) calcd for C_24_H_29_N_7_O: 431.2433; found: 431.2435.

*N^1^-((4-Fluorophenyl)(tert-butyl-1H-tetrazol-5-yl)methyl)-N^2^-(6-methoxyquinolin-8-yl)ethan-1,2-diamine* (**9**): The reaction of compound **6** (163 mg (0.75 mmol)), 4-fluorobenzaldehyde (93 mg, 81 µL (0.75 mmol)), trimethylsilyl azide (86 mg, 100 µL (0.75 mmol)) and *tert*-butyl isocyanide (62 mg, 85 µL (2.00 mmol)) in dry methanol (5 mL) gave the raw tetrazole which was purified by column chromatography (silica gel, diethyl ether) to yield compound **9** as yellow oil (206 mg (61%)). IR = 2938, 1619, 1578, 1520, 1459, 1423, 1388, 1215, 1156, 1109, 824, 792; ^1^H NMR (CDCl_3_, 400 MHz) *δ* = 1.57 (s, 9H, (CH_3_)_3_), 2.44 (br, 1H, NH), 2.89 (dt, *J* = 12.2, 6.1 Hz, 1H, 2′-H), 2.99 (dt, *J* = 12.2, 6.1 Hz, 1H, 2′-H), 3.38–3.43 (m, 2H, 1′-H), 3.88 (s, 3H, OCH_3_), 5.36 (s, 1H, 1″-H), 6.29 (d, *J* = 2.5 Hz, 1H, 7-H), 6.38 (d, *J* = 2.5 Hz, 1H, 5-H), 6.49 (br, 1H, NH), 6.98–7.03 (m, 2H, aromatic H), 7.27–7.34 (m, 3H, 3-H, aromatic H), 7.94 (dd, *J* = 8.2, 1.6 Hz, 1H, 4-H), 8.55 (dd, *J* = 4.2, 1.6 Hz, 1H, 2-H); ^13^C NMR (CDCl_3_, 100 MHz) *δ* = 29.92 ((CH_3_)_3_), 43.09 (C-1′), 46.21 (C-2′), 55.20 (OCH_3_), 58.03 (C-1″), 61.37 (C*Me*_3_), 92.39 (C-5), 97.04 (C-7), 115.81 (d, *J* = 21.8 Hz, aromatic C), 121.90 (C-3), 129.73 (C-4a), 129.89 (d, *J* = 8.4 Hz, aromatic C), 134.56 (d, *J* = 3.5 Hz, aromatic C_q_), 134.68 (C-4), 135.44 (C-8a), 144.53 (C-2), 145.66 (C-8), 155.45 (C-5‴), 159.32 (C-6), 162.49 (d, *J* = 248 Hz, aromatic C_q_); HRMS (EI+) calcd for C_24_H_28_FN_7_O: 449.2339; found: 449.2348.

*N^1^-((4-Chlorophenyl)(tert-butyl-1H-tetrazol-5-yl)methyl)-N^2^-(6-methoxyquinolin-8-yl)ethan-1,2-diamine* (**10**): The reaction of compound **6** (163 mg (0.75 mmol)), 4-chlorobenzaldehyde (105 mg (0.75 mmol)), trimethylsilyl azide (86 mg, 100 µL (0.75 mmol)) and *tert*-butyl isocyanide (62 mg, 85 µL (2.00 mmol)) in dry methanol (5 mL) gave the raw tetrazole which was purified by column chromatography (silica gel, diethyl ether) to yield compound **10** as yellow oil (73 mg (21%)). IR = 2938, 1619, 1578, 1520, 1458, 1423, 1388, 1213, 1154, 1091, 1014, 821, 791, 730; ^1^H NMR (CDCl_3_, 400 MHz) *δ* = 1.57 (s, 9H, (CH_3_)_3_), 2.46 (br, 1H, NH), 2.88 (dt, *J* = 12.0, 6.0 Hz, 1H, 2′-H), 2.99 (dt, *J* = 12.0, 6.0 Hz, 1H, 2′-H), 3.37–3.42 (m, 2H, 1′-H), 3.88 (s, 3H, OCH_3_), 5.35 (s, 1H, 1″-H), 6.29 (d, *J* = 2.0 Hz, 1H, 7-H), 6.37 (d, *J* = 2.0 Hz, 1H, 5-H), 6.49 (br, 1H, NH), 7.25 (d, *J* = 8.5 Hz, 2H, aromatic H), 7.29 (d, *J* = 8.5 Hz, 2H, aromatic H), 7.31 (dd, *J* = 8.3, 4.1 Hz, 1H, 3-H), 7.93 (dd, *J* = 8.3, 1.6 Hz, 1H, 4-H), 8.55 (dd, *J* = 4.1 Hz, 1H, 2-H); ^13^C NMR (CDCl_3_, 100 MHz) *δ* = 29.95 ((CH_3_)_3_), 43.06 (C-1′), 46.12 (C-2′), 55.22 (OCH_3_), 58.08 (C-1″), 61.41 (C*Me*_3_), 92.41 (C-5), 97.07 (C-7), 121.91 (C-3), 129.06, 129.51 (aromatic C), 129.73 (C-4a), 134.31 (aromatic C_q_), 134.69 (C-4), 135.44 (C-8a), 137.22 (aromatic C_q_), 144.54 (C-2), 145.64 (C-8), 155.23 (C-5‴), 159.32 (C-6); HRMS (EI+) calcd for C_24_H_28_ClN_7_O: 465.2044; found: 465.2051.

*N^1^-((4-Bromophenyl)(tert-butyl-1H-tetrazol-5-yl)methyl)-N^2^-(6-methoxyquinolin-8-yl)ethan-1,2-diamine* (**11**): The reaction of compound **6** (163 mg (0.75 mmol)), 4-bromobenzaldehyde (139 mg (0.75 mmol)), trimethylsilyl azide (86 mg, 100 µL (0.75 mmol)) and *tert*-butyl isocyanide (62 mg, 85 µL (2.00 mmol)) in dry methanol (5 mL) gave the raw tetrazole which was purified by column chromatography (silica gel, diethyl ether) to yield compound **11** as yellow oil (123 mg (32%)). IR = 2936, 1619, 1578, 1520, 1458, 1388, 1213, 1154, 1107, 1010, 821, 791; ^1^H NMR (CDCl_3_, 400 MHz) *δ* = 1.58 (s, 9H, (CH_3_)_3_), 2.46 (br, 1H, NH), 2.89 (dt, *J* = 12.0, 6.0 Hz, 1H, 2′-H), 2.99 (dt, *J* = 12.0, 6.0 Hz, 1H, 2′-H), 3.37–3.42 (m, 2H, 1′-H), 3.89 (s, 3H, OCH_3_), 5.34 (s, 1H, 1″-H), 6.29 (d, *J* = 2.5 Hz, 1H, 7-H), 6.38 (d, *J* = 2.5 Hz, 1H, 5-H), 6.50 (br, 1H, NH), 7.19 (d, *J* = 8.4 Hz, 2H, aromatic H), 7.32 (dd, *J* = 8.2, 4.2 Hz, 1H, 3-H), 7.44 (d, *J* = 8.4 Hz, 2H, aromatic H), 7.94 (dd, *J* = 8.2, 1.6 Hz, 1H, 4-H), 8.55 (dd, *J* = 4.2, 1.6 Hz, 1H, 2-H); ^13^C NMR (CDCl_3_, 100 MHz) *δ* = 29.99 ((CH_3_)_3_), 43.09 (C-1′), 46.23 (C-2′), 55.25 (OCH_3_), 58.18 (C-1″), 61.44 (C*Me*_3_), 92.44 (C-5), 97.11 (C-7), 121.94 (C-3), 122.52 (aromatic C_q_), 129.76 (C-4a), 129.85, 132.06 (aromatic C), 134.73 (C-4), 135.45 (C-8a), 137.75 (aromatic C_q_), 144.56 (C-2), 145.65 (C-8), 155.18 (C-5‴), 159.34 (C-6); HRMS (EI+) calcd for C_24_H_28_BrN_7_O: 509.1539; found: 509.1533.

*N^1^-(6-Methoxyquinolin-8-yl)-N^2^-((tert-butyl-1H-tetrazol-5-yl)(2-(trifluoromethyl)phenyl) methyl)-ethan-1,2-diamine* (**12**): The reaction of compound **6** (163 mg (0.75 mmol)), 4-(trifluoromethyl)benzaldehyde (131 mg, 102 µL (0.75 mmol)), trimethylsilyl azide (86 mg, 100 µL (0.75 mmol)) and *tert*-butyl isocyanide (62 mg, 85 µL (2.00 mmol)) in dry methanol (5 mL) gave the raw tetrazole which was purified by column chromatography (silica gel, diethyl ether) to yield compound **12** as yellow oil (56 mg (15%)). IR = 2937, 1619, 1579, 1521, 1457, 1424, 1389, 1335, 1313, 1264, 1214, 1164, 1115, 1036, 823, 771; ^1^H NMR (CDCl_3_, 400 MHz) *δ* = 1.73 (s, 9H, (CH_3_)_3_), 2.26 (br, 1H, NH), 2.93 (t, *J* = 6.0 Hz, 2H, 2′-H), 3.32–3.46 (m, 2H, 1′-H), 3.87 (s, 3H, OCH_3_), 5.84 (s, 1H, 1″-H), 6.25 (d, *J* = 2.5 Hz, 1H, 7-H), 6.37 (d, *J* = 2.5 Hz, 1H, 5-H), 6.38–6.41 (m, 1H, NH), 7.31 (dd, *J* = 8.3, 4.2 Hz, 1H, 3-H), 7.44 (t, *J* = 7.6 Hz, 1H, aromatic H), 7.55 (t, *J* = 7.5 Hz, 1H, aromatic H), 7.61 (d, *J* = 7.5 Hz, 1H, aromatic H), 7.71 (d, *J* = 7.6 Hz, 1H, aromatic H), 7.93 (dd, *J* = 8.3, 1.6 Hz, 1H, 4-H), 8.52 (dd, *J* = 4.2, 1.6 Hz, 1H, 2-H); ^13^C NMR (CDCl_3_, 100 MHz) *δ* = 29.75 ((CH_3_)_3_), 42.94 (C-1′), 46.78 (C-2′), 53.81 (C-1″), 55.21 (OCH_3_), 62.38 (C*Me*_3_), 92.42 (C-5), 96.91 (C-7), 121.91 (C-3), 124.44 (q, *J* = 274 Hz, CF_3_), 126.51 (q, *J* = 5.9 Hz, aromatic C), 128.34 (aromatic C), 128.42 (q, *J* = 30.1 Hz, aromatic C_q_), 129.24 (aromatic C), 129.68 (C-4a), 132.45 (aromatic C), 134.65 (C-4), 135.40 (C-8a), 136.75 (aromatic C_q_), 144.48 (C-2), 145.57 (C-8), 154.65 (C-5‴), 159.29 (C-6); HRMS (EI+) calcd for C_25_H_28_F_3_N_7_O: 499.2307; found: 499.2317.

*6-Methoxy-N-(1-(1-tert-butyl-1H-tetrazol-5-yl)propyl)quinolin-8-amine* (**13**): The reaction of 6-methoxyquinolin-8-amine **2** (131 mg (0.75 mmol)), propanal (44 mg, 54 µL (0.75 mmol)), trimethylsilyl azide (86 mg, 100 µL (0.75 mmol)) and *tert*-butyl isocyanide (62 mg, 85 µL (2.00 mmol)) in dry methanol (5 mL) gave the raw tetrazole which was purified by column chromatography (silica gel, CH_2_Cl_2_/*Me*OH 80:1) to yield compound **13** as yellow oil (107 mg (42%)). IR = 2966, 2937, 1620, 1596, 1579, 1520, 1454, 1422, 1391, 1339, 1324, 1220, 1195, 1169, 1125, 1064, 1050, 1031, 899, 838, 820, 792; ^1^H NMR (CDCl_3_, 400 MHz) *δ* = 1.01 (t, *J* = 7.4 Hz, 3H, 3′-H), 1.76 (s, 9H, (CH_3_)_3_), 2.21–2.41 (m, 2H, 2′-H), 3.91 (s, 3H, OCH_3_), 5.08 (td, *J* = 9.0, 5.5 Hz, 1H, 1′-H), 6.37 (d, *J* = 1.6 Hz, 1H, 7-H), 6.43 (d, *J* = 1.6 Hz, 1H, 5-H), 6.63 (d, *J* = 9.0 Hz, 1H, NH), 7.33 (dd, *J* = 8.3, 4.2 Hz, 1H, 3-H), 7.94 (dd, *J* = 8.3, 1.6 Hz, 1H, 4-H), 8.54 (dd, *J* = 4.2, 1.6 Hz, 1H, 2-H); ^13^C NMR (CDCl_3_, 100 MHz) *δ* = 10.82 (C-3′), 26.98 (C-2′), 30.10 ((CH_3_)_3_), 50.43 (C-1′), 55.31 (OCH_3_), 61.53 (C*Me*_3_), 93.11 (C-5), 97.25 (C-7), 122.24 (C-3), 129.80 (C-4a), 134.69 (C-4), 135.34 (C-8a), 143.57 (C-8), 144.99 (C-2), 155.09 (C-5″), 158.86 (C-6); HRMS (EI+) calcd for C_18_H_24_N_6_O: 340.2012; found: 340.2024.

*6-Methoxy-N-((phenyl)(1-tert-butyl-1H-tetrazol-5-yl)methyl)quinolin-8-amine* (**14**): The reaction of 6-methoxyquinolin-8-amine **2** (131 mg (0.75 mmol)), benzaldehyde (80 mg, 76 µL (0.75 mmol)), trimethylsilyl azide (86 mg, 100 µL (0.75 mmol)) and *tert*-butyl isocyanide (62 mg, 85 µL (2.00 mmol)) in dry methanol (5 mL) gave the raw tetrazole which was purified by column chromatography (silica gel, CH_2_Cl_2_/*Me*OH 80:1) to yield compound **14** as yellow oil (64 mg (22%)). IR = 2978, 2938, 1624, 1596, 1580, 1519, 1494, 1453, 1421, 1392, 1337, 1216, 1163, 1066, 1025, 824, 789, 735; ^1^H NMR (CDCl_3_, 400 MHz) *δ* = 1.75 (s, 9H, (CH_3_)_3_), 3.82 (s, 3H, OCH_3_), 6.24 (d, *J* = 2.3 Hz, 1H, 7-H), 6.28 (d, *J* = 8.4 Hz, 1H, 1′-H), 6.40 (d, *J* = 2.3 Hz, 1H, 5-H), 7.20 (d, *J* = 8.4 Hz, 1H, NH), 7.29 (m, 4H, 3-H, aromatic H), 7.46 (d, *J* = 7.0 Hz, 2H, aromatic H), 7.92 (dd, *J* = 8.3, 1.6 Hz, 1H, 4-H), 8.56 (dd, *J* = 4.2, 1.6 Hz, 1H, 2-H); ^13^C NMR (CDCl_3_, 100 MHz) *δ* = 30.06 ((CH_3_)_3_), 53.25 (C-1′), 55.17 (OCH_3_), 61.78 (C*Me*_3_), 93.62 (C-5), 98.33 (C-7), 122.15 (C-3), 127.65, 128.56, 129.00 (aromatic C), 129.62 (C-4a), 134.62 (C-4), 135.26 (C-8a), 137.56 (aromatic C_q_), 143.17 (C-8), 145.05 (C-2), 154.95 (C-5″), 158.63 (C-6); HRMS (EI+) calcd for C_22_H_24_N_6_O: 388.2012; found: 388.2027.

*N-((4-Fluorophenyl)(1-tert-butyl-1H-tetrazol-5-yl)methyl)-6-methoxyquinolin-8-amine* (**15**): The reaction of 6-methoxyquinolin-8-amine **2** (131 mg (0.75 mmol)), 4-fluorobenzaldehyde (93 mg, 81 µL (0.75 mmol)), trimethylsilyl azide (86 mg, 100 µL (0.75 mmol)) and *tert*-butyl isocyanide (62 mg, 85 µL (2.00 mmol)) in dry methanol (5 mL) gave the raw tetrazole which was purified by column chromatography (silica gel, diethyl ether) to yield compound **15** as orange oil (95 mg (31%)). IR = 2985, 1622, 1579, 1512, 1454, 1422, 1389, 1337, 1218, 1160, 826, 791; ^1^H NMR (CDCl_3_, 400 MHz) *δ* = 1.76 (s, 9H, (CH_3_)_3_), 3.84 (s, 3H, OCH_3_), 6.22 (d, *J* = 2.5 Hz, 1H, 7-H), 6.25 (d, *J* = 8.4 Hz, 1H, 1′-H), 6.43 (d, *J* = 2.5 Hz, 1H, 5-H), 7.02–7.07 (m, 2H, aromatic H), 7.16 (d, *J* = 8.4 Hz, 1H, NH), 7.34 (dd, *J* = 8.3, 4.2 Hz, 1H, 3-H), 7.43–7.48 (m, 2H, aromatic H), 7.93 (dd, *J* = 8.3, 1.5 Hz, 1H, 4-H), 8.57 (dd, *J* = 4.2, 1.5 Hz, 1H, 2-H); ^13^C NMR (CDCl_3_, 100 MHz) *δ* = 30.12 ((CH_3_)_3_), 52.63 (C-1′), 55.25 (OCH_3_), 61.90 (C*Me*_3_), 93.84 (C-5), 98.54 (C-7), 116.03 (d, *J* = 21.8 Hz, aromatic C), 129.41 (d, *J* = 8.4 Hz, aromatic C), 129.68 (C-4a), 133.43 (d, *J* = 2.8 Hz, aromatic C), 134.70 (C-4), 135.28 (C-8a), 142.98 (C-8), 145.19 (C-2), 154.86 (C-5″), 158.65 (C-6), 162.68 (d, *J* = 248 Hz, aromatic C_q_); HRMS (EI+) calcd for C_22_H_23_FN_6_O: 406.1917; found: 406.1923.

*N-((4-Chlorophenyl)(1-tert-butyl-1H-tetrazol-5-yl)methyl)-6-methoxyquinolin-8-amine* (**16**): The reaction of 6-methoxyquinolin-8-amine **2** (131 mg (0.75 mmol)), 4-chlorobenzaldehyde (105 mg (0.75 mmol)), trimethylsilyl azide (86 mg, 100 µL (0.75 mmol)) and *tert*-butyl isocyanide (62 mg, 85 µL (2.00 mmol)) in dry methanol (5 mL) gave the raw tetrazole which was purified by column chromatography (silica gel, diethyl ether) followed by precipitation from *Me*OH to yield compound **16** as off-white amorphous solid (19 mg (6%)). IR = 2992, 1625, 1579, 1515, 1493, 1454, 1420, 1386, 1220, 1168, 1113, 1015, 822, 790; ^1^H NMR (CDCl_3_, 400 MHz) *δ* = 1.77 (s, 9H, (CH_3_)_3_), 3.84 (s, 3H, OCH_3_), 6.22 (d, *J* = 2.5 Hz, 1H, 7-H), 6.24 (d, *J* = 8.4 Hz, 1H, 1′-H), 6.43 (d, *J* = 2.5 Hz, 1H, 5-H), 7.15 (d, *J* = 8.4 Hz, 1H, NH), 7.32 (d, *J* = 8.5 Hz, 2H, aromatic H), 7.33 (dd, *J* = 8.3, 4.2 Hz, 1H, 3-H), 7.42 (d, *J* = 8.5 Hz, 2H, aromatic H), 7.93 (dd, *J* = 8.3, 1.6 Hz, 1H, 4-H), 8.56 (dd, *J* = 4.2, 1.6 Hz, 1H, 2-H); ^13^C NMR (CDCl_3_, 100 MHz) *δ* = 30.12 ((CH_3_)_3_), 52.68 (C-1′), 55.24 (OCH_3_), 61.95 (C*Me*_3_), 93.93 (C-5), 98.60 (C-7), 122.29 (C-3), 128.98, 129.23 (aromatic C), 129.68 (C-4a), 134.49 (aromatic C_q_), 134.70 (C-4), 135.24 (C-8a), 136.19 (aromatic C_q_), 142.89 (C-8), 145.20 (C-2), 154.63 (C-5″), 158.64 (C-6); HRMS (EI+) calcd for C_22_H_23_ClN_6_O: 422.1622; found: 422.1646.

*N-((4-Bromophenyl)(1-tert-butyl-1H-tetrazol-5-yl)methyl)-6-methoxyquinolin-8-amine* (**17**): The reaction of 6-methoxyquinolin-8-amine **2** (131 mg (0.75 mmol)), 4-bromobenzaldehyde (139 mg (0.75 mmol)), trimethylsilyl azide (86 mg, 100 µL (0.75 mmol)) and *tert*-butyl isocyanide (62 mg, 85 µL (2.00 mmol)) in dry methanol (5 mL) gave the raw tetrazole which precipitated from diethyl ether to yield compound **17** as off-white amorphous solid (112 mg (32%)). IR = 2984, 1622, 1579, 1517, 1488, 1454, 1422, 1389, 1216, 1164, 1072, 1011, 824, 791; ^1^H NMR (CDCl_3_, 400 MHz) *δ* = 1.77 (s, 9H, (CH_3_)_3_), 3.84 (s, 3H, OCH_3_), 6.21 (d, *J* = 2.3 Hz, 1H, 7-H), 6.22 (d, *J* = 8.4 Hz, 1H, 1′-H), 6.43 (d, *J* = 2.3 Hz, 1H, 5-H), 7.15 (d, *J* = 8.4 Hz, 1H, NH), 7.34 (dd, *J* = 8.4, 4.4 Hz, 1H, 3-H), 7.36 (d, *J* = 8.4 Hz, 2H, aromatic H), 7.48 (d, *J* = 8.4 Hz, 2H, aromatic H), 7.94 (dd, *J* = 8.4, 1.3 Hz, 1H, 4-H), 8.56 (dd, *J* = 4.4, 1.3 Hz, 1H, 2-H); ^13^C NMR (CDCl_3_, 100 MHz) *δ* = 30.15 ((CH_3_)_3_), 52.75 (C-1′), 55.26 (OCH_3_), 61.97 (C*Me*_3_), 93.94 (C-5), 98.62 (C-7), 122.31 (C-3), 122.69 (aromatic C_q_), 129.30 (aromatic C), 129.70 (C-4a), 132.19 (aromatic C), 134.71 (C-4), 135.25 (C-8a), 136.73 (aromatic C_q_), 142.88 (C-8), 145.22 (C-2), 154.55 (C-5″), 158.65 (C-6); HRMS (EI+) calcd for C_22_H_23_BrN_6_O: 466.1117; found: 466.1137.

*6-Methoxy-N-((1-tert-butyl-1H-tetrazol-5-yl)(4-(trifluoromethyl)phenyl)methyl)quinolin-8-amine* (**18**): The reaction of 6-methoxyquinolin-8-amine **2** (131 mg (0.75 mmol)), 4-(trifluoromethyl) benzaldehyde (131 mg, 102 µL (0.75 mmol)), trimethylsilyl azide (86 mg, 100 µL (0.75 mmol)) and *tert*-butyl isocyanide (62 mg, 85 µL (2.00 mmol)) in dry methanol (5 mL) gave the raw tetrazole which was purified by column chromatography (silica gel, diethyl ether/petroleum ether 20:1) followed by precipitation from diethyl ether to yield compound **18** as off-white amorphous solid (17 mg (5%)). IR = 2936, 1622, 1580, 1515, 1454, 1421, 1388, 1325, 1220, 1168, 1112, 1068, 1018, 823, 790; ^1^H NMR (CDCl_3_, 400 MHz) *δ* = 1.79 (s, 9H, (CH_3_)_3_), 3.84 (s, 3H, OCH_3_), 6.22 (d, *J* = 2.4 Hz, 1H, 7-H), 6.33 (d, *J* = 8.5 Hz, 1H, 1′-H), 6.45 (d, *J* = 2.4 Hz, 1H, 5-H), 7.18 (d, *J* = 8.5 Hz, 1H, NH), 7.36 (dd, *J* = 8.2, 4.2 Hz, 1H, 3-H), 7.61 (s, 4H, aromatic H), 7.95 (dd, *J* = 8.2, 1.4 Hz, 1H, 4-H), 8.57 (dd, *J* = 4.2, 1.4 Hz, 1H, 2-H); ^13^C NMR (CDCl_3_, 100 MHz) *δ* = 30.15 ((CH_3_)_3_), 52.79 (C-1′), 55.27 (OCH_3_), 62.08 (C*Me*_3_), 94.09 (C-5), 98.61 (C-7), 122.38 (C-3), 123.84 (q, *J* = 272 Hz, CF_3_), 126.02 (q, *J* = 3.8 Hz, aromatic C), 127.94 (aromatic C), 129.72 (C-4a), 130.76 (q, *J* = 33.0 Hz, aromatic C_q_), 134.76 (C-4), 135.17 (C-8a), 141.64 (aromatic C_q_), 142.73 (C-8), 145.29 (C-2), 154.33 (C-5″), 158.63 (C-6); HRMS (EI+) calcd for C_23_H_23_F_3_N_6_O: 456.1885; found: 456.1904.

*6-Methoxy-N-((4-methylphenyl)(1-tert-butyl-1H-tetrazol-5-yl)methyl)quinolin-8-amine* (**19**): The reaction of 6-methoxyquinolin-8-amine **2** (131 mg (0.75 mmol)), 4-methylbenzaldehyde (90 mg, 88 µL (0.75 mmol)), trimethylsilyl azide (86 mg, 100 µL (0.75 mmol)) and *tert*-butyl isocyanide (62 mg, 85 µL (2.00 mmol)) in dry methanol (5 mL) gave the raw tetrazole which was purified by column chromatography (silica gel, diethyl ether) followed by precipitation from *Me*OH to yield compound **19** as off-white amorphous solid (51 mg (17%)). IR = 2985, 1622, 1579, 1517, 1454, 1422, 1389, 1216, 1165, 1025, 824, 791; ^1^H NMR (CDCl_3_, 400 MHz) *δ* = 1.75 (s, 9H, (CH_3_)_3_), 2.32 (s, 3H, CH_3_), 3.83 (s, 3H, OCH_3_), 6.23 (d, *J* = 2.3 Hz, 1H, 7-H), 6.24 (d, *J* = 8.4 Hz, 1H, 1′-H), 6.40 (d, *J* = 2.3 Hz, 1H, 5-H), 7.15 (d, *J* = 7.9 Hz, 2H, aromatic H), 7.19 (d, *J* = 8.4 Hz, 1H, NH), 7.33 (dd, *J* = 8.1, 4.2 Hz, 1H, 3-H), 7.34 (d, *J* = 7.9 Hz, 2H, aromatic H), 7.92 (dd, *J* = 8.1, 1.6 Hz, 1H, 4-H), 8.56 (dd, *J* = 4.2, 1.6 Hz, 1H, 2-H); ^13^C NMR (CDCl_3_, 100 MHz) *δ* = 21.13 (CH_3_), 30.12 ((CH_3_)_3_), 53.12 (C-1′), 55.21 (OCH_3_), 61.77 (C*Me*_3_), 93.58 (C-5), 98.33 (C-7), 122.16 (C-3), 127.64 (aromatic C), 129.67 (C-4a), 129.74 (aromatic C), 134.61 (C-4), 134.65 (aromatic C_q_), 135.40 (C-8a), 138.43 (aromatic C_q_), 143.34 (C-8), 145.08 (C-2), 155.14 (C-5″), 158.73 (C-6); HRMS (EI+) calcd for C_23_H_26_N_6_O: 402.2168; found: 402.2182.

*6-Methoxy-N-((4-(propan-2-yl)phenyl)(1-tert-butyl-1H-tetrazol-5-yl)methyl)quinolin-8-amine* (**20**): The reaction of 6-methoxyquinolin-8-amine **2** (131 mg (0.75 mmol)), 4-(propan-2-yl)benzaldehyde (93 mg, 81 µL (0.75 mmol)), trimethylsilyl azide (86 mg, 100 µL (0.75 mmol)) and *tert*-butyl isocyanide (62 mg, 85 µL (2.00 mmol)) in dry methanol (5 mL) gave the raw tetrazole which was purified by column chromatography (silica gel, diethyl ether) to yield compound **20** as orange oil (26 mg (8%)). IR = 2961, 1622, 1578, 1517, 1454, 1422, 1388, 1216, 1165, 1054, 1024, 906, 824, 730; ^1^H NMR (CDCl_3_, 400 MHz) *δ* = 1.21 (d, *J* = 6.9 Hz, 6H, (CH_3_)_2_), 1.75 (s, 9H, (CH_3_)_3_), 2.87 (sept, *J* = 6.9 Hz, 1H, C*H*(CH_3_)_2_), 3.84 (s, 3H, OCH_3_), 6.24 (d, *J* = 2.3 Hz, 1H, 7-H), 6.25 (d, *J* = 8.4 Hz, 1H, 1′-H), 6.40 (d, *J* = 2.3 Hz, 1H, 5-H), 7.19 (d, *J* = 8.4 Hz, 1H, NH), 7.20 (d, *J* = 8.1 Hz, 2H, aromatic H), 7.31 (dd, *J* = 8.3, 4.2 Hz, 1H, 3-H), 7.36 (d, *J* = 8.1 Hz, 2H, aromatic H), 7.92 (dd, *J* = 8.3, 1.5 Hz, 1H, 4-H), 8.56 (dd, *J* = 4.2, 1.5 Hz, 1H, 2-H); ^13^C NMR (CDCl_3_, 100 MHz) *δ* = 23.83 ((CH_3_)_2_), 30.12 ((CH_3_)_3_), 33.75 (*C*H(CH_3_)_2_), 53.07 (C-1′), 55.21 (OCH_3_), (C*Me*_3_), 93.53 (C-5), 98.20 (C-7), 122.15 (C-3), 127.11, 127.66 (aromatic C), 129.67 (C-4a), 134.61 (C-4), 134.92 (aromatic C_q_), 135.37 (C-8a), 143.40 (C-8), 145.06 (C-2), 149.24 (aromatic C_q_), 155.18 (C-5″), 158.72 (C-6); HRMS (EI+) calcd for C_25_H_30_N_6_O: 430.2481; found: 430.2512.

*6-Methoxy-N-((naphthalen-1-yl)(1-tert-butyl-1H-tetrazol-5-yl)methyl)quinolin-8-amine* (**21**): The reaction of 6-methoxyquinolin-8-amine **2** (131 mg (0.75 mmol)), naphthalene-1-carboxaldehyde (117 mg, 102 µL (0.75 mmol)), trimethylsilyl azide (86 mg, 100 µL (0.75 mmol)) and *tert*-butyl isocyanide (62 mg, 85 µL (2.00 mmol)) in dry methanol (5 mL) gave the raw tetrazole which was purified by column chromatography (silica gel, diethyl ether) followed by precipitation from *Me*OH to yield compound **21** as off-white amorphous solid (82 mg (25%)). IR = 2937, 1619, 1578, 1518, 1452, 1422, 1389, 1278, 1216, 1160, 1047, 1029, 825, 781, 738; ^1^H NMR (CDCl_3_, 400 MHz) *δ* = 1.68 (s, 9H, (CH_3_)_3_), 3.83 (s, 3H, OCH_3_), 6.23 (d, *J* = 2.4 Hz, 1H, 7-H), 6.43 (d, *J* = 2.4 Hz, 1H, 5-H), 6.99 (d, *J* = 9.0 Hz, 1H, 1′-H), 7.09 (d, *J* = 9.0 Hz, 1H, NH), 7.18 (d, *J* = 7.2 Hz, 1H, aromatic H), 7.29 (dd, *J* = 8.3, 4.2 Hz, 1H, 3-H), 7.39 (t, *J* = 7.7 Hz, 1H, aromatic H), 7.48–7.53 (m, 2H, aromatic H), 7.85–7.98 (m, 4H, 4-H, aromatic H), 8.50 (dd, *J* = 4.2, 1.4 Hz, 1H, 2-H); ^13^C NMR (CDCl_3_, 100 MHz) *δ* = ((CH_3_)_3_), 50.32 (C-1′), 55.17 (OCH_3_), 62.10 (C*Me*_3_), 93.80 (C-5), 98.15 (C-7), 122.16 (C-3), 122.23, 125.35, 125.91, 126.12, 127.29, 129.16, 129.72 (aromatic C), 129.81 (C-4a), 130.94, 132.97, 134.11 (aromatic C_q_), 135.44 (C-8a), 143.61 (C-8), 145.08 (C-2), 154.86 (C-5″), 158.70 (C-6); HRMS (EI+) calcd for C_26_H_26_N_6_O: 438.2168; found: 438.2200.

*6-Methoxy-N-(1-(1-tert-butyl-1H-tetrazol-5-yl)octyl)quinolin-8-amine* (**22**): The reaction of 6-methoxyquinolin-8-amine **2** (131 mg (0.75 mmol)), octanal (96 mg, 117 µL (0.75 mmol)), trimethylsilyl azide (86 mg, 100 µL (0.75 mmol)) and *tert*-butyl isocyanide (62 mg, 85 µL (2.00 mmol)) in dry methanol (5 mL) gave the raw tetrazole which was purified by column chromatography (silica gel, diethyl ether/cyclohexane 1:1) to yield compound **22** as light brown oil (99 mg (30%)). IR = 2928, 2855, 1621, 1579, 1519, 1455, 1422, 1389, 1216, 1196, 1166, 1050, 824, 792; ^1^H NMR (CDCl_3_, 400 MHz) *δ* = 0.85 (t, *J* = 7.0 Hz, 3H, 8′-H), 1.17–1.47 (m, 10H, 3′-H, 4′-H, 5′-H, 6′-H, 7′-H), 1.76 (s, 9H, (CH_3_)_3_), 2.15–2.37 (m, 2H, 2′-H), 3.91 (s, 3H, OCH_3_), 5.10–5.17 (m, 1H, 1′-H), 6.36 (d, *J* = 1.9 Hz, 1H, 7-H), 6.43 (d, *J* = 1.9 Hz, 1H, 5-H), 6.62 (d, *J* = 9.7 Hz, 1H, NH), 7.31 (dd, *J* = 8.2, 4.1 Hz, 1H, 3-H), 7.93 (dd, *J* = 8.2, 1.5 Hz, 1H, 4-H), 8.53 (dd, *J* = 4.1, 1.5 Hz, 1H, 2-H); ^13^C NMR (CDCl_3_, 100 MHz) *δ* = 13.97 (C-8′), 22.52 (C-7′), 26.32 (C-3′), 29.02 (C-5′), 29.33 (C-4′), 30.08 ((CH_3_)_3_), 31.66 (C-6′), 34.03 (C-2′), 49.16 (C-1′), 55.26 (OCH_3_), 61.48 (C*Me*_3_), 93.14 (C-5), 97.23 (C-7), 122.16 (C-3), 129.79 (C-4a), 134.64 (C-4), 135.33 (C-8a), 143.56 (C-8), 144.92 (C-2), 155.36 (C-5″), 158.87 (C-6); HRMS (EI+) calcd for C_23_H_34_N_6_O: 410.2794; found: 410.2793.

### 3.3. Biological Tests

#### 3.3.1. In Vitro Microplate Assay against *P. falciparum*

In vitro activity against erythrocytic stages of *P. falciparum* was determined using a ^3^H-hypoxanthine incorporation assay [22,23], using the drug-sensitive NF54 strain [24]. Chloroquine (Sigma C6628) was used as standard. Compounds were dissolved in DMSO at 10 mg/mL and added to parasite cultures incubated in RPMI 1640 medium without hypoxanthine, supplemented with HEPES (5.94 g/L), NaHCO_3_ (2.1 g/L), neomycin (100 U/mL), Albumax (5 g/L) and washed human red cells A^+^ at 2.5% hematocrit (0.3% parasitemia). Serial drug dilutions of 11 3-fold dilution steps covering a range from 100 to 0.002 μg/mL were prepared. The 96-well plates were incubated in a humidified atmosphere at 37 °C; 4% CO_2_, 3% O_2_, 93% N_2_. After 48 h, 0.05 mL of ^3^H-hypoxanthine (=0.5 μCi) was added to each well of the plate. The plates were incubated for a further 24 h under the same conditions. The plates were then harvested with a Betaplate cell harvester (Wallac, Zurich, Switzerland). The red blood cells were transferred onto a glass fiber filter and washed with distilled water. The dried filters were inserted into a plastic foil with 10 mL of scintillation fluid and counted in a Betaplate liquid scintillation counter (Wallac, Zurich, Switzerland). IC_50_ values were calculated from sigmoidal inhibition curves by linear regression [25] using Microsoft Excel. Artemisinin and chloroquine were used as control.

#### 3.3.2. In Vitro Cytotoxicity with L-6 Cells

Assays were performed in 96-well microtiter plates, each well containing 0.1 mL of RPMI 1640 medium supplemented with 1% L-glutamine (200 mM) and 10% fetal bovine serum and 4000 L-6 cells (a primary cell line derived from rat skeletal myoblasts) [26,27]. Serial drug dilutions of 11 3-fold dilution steps covering a range from 100 to 0.002 μg/mL were prepared. After 70 h of incubation, the plates were inspected under an inverted microscope to assure the growth of the controls and sterile conditions. Then, 0.01 mL of Alamar Blue was added to each well and the plates incubated for another 2 h. The plates were read with a Spectramax Gemini XS microplate fluorometer (Molecular Devices Cooperation, Sunnyvale, CA, USA) using an excitation wavelength of 536 nm and an emission wavelength of 588 nm. The IC_50_ values were calculated by linear regression [28] from the sigmoidal dose inhibition curves using SoftmaxPro software (Molecular Devices Cooperation, Sunnyvale, CA, USA). Podophyllotoxin (Sigma P4405) was used as control.

## 4. Conclusions

A series of 8-amino-6-methoxyquinoline hybrids was prepared via Ugi-azide reaction. The compounds exhibit different linkers between a 6-methoxyquinolin-8-amine moiety and a tetrazole ring. Compounds with a short and non-basic linker with lipophilic substitution showed the highest antiplasmodial activity. Six of the new derivates have promising selectivity due to their low cytotoxity. The optimum linker length will be investigated in a future project.

## Figures and Tables

**Table 1:** Activities of compounds **7**–**22** against *P. falciparum* NF54, and L-6 cells, expressed as IC_50_ (µM) ^a^.

Compound	*P.f.* NF54 ^b^ IC_50_ (µM)	S.I. = IC_50_ (Cyt.)/IC_50_ (*P.f.* NF54)	Cytotoxicity L-6 Cells IC_50_ (µM)
**7**	15.98	15.98	144.0
**8**	6.09	9.52	57.97
**9**	7.05	7.86	55.43
**10**	5.34	17.29	92.31
**11**	2.51	13.57	34.07
**12**	2.92	7.64	22.30
**13**	23.60	3.86	91.10
**14**	5.12	9.56	48.96
**15**	2.68	46.27	124.0
**16**	0.743	318.3	236.5
**17**	0.464	208.8	96.86
**18**	0.644	273.3	176.0
**19**	1.04	238.9	248.5
**20**	2.00	59.70	119.4
**21**	1.26	137.6	173.4
**22**	0.324	168.9	54.73
**CQ**	0.0039	23,313	90.92
**POD**			0.012

CQ = chloroquine; POD = podophyllotoxin. ^a^ values represent the average of four determinations (two determinations of two independent experiments); ^b^ sensitive to chloroquine.

## Data Availability

The data presented in this study are available in this article.

## Supplementary Materials

The following are available online. Figures S1–S20: ^1^H- and ^13^C-NMR spectra for compounds **3**–**22** and IR and MS spectra for compounds **7**–**22**.

## Sample Availability

Samples of the compounds are available from the authors.
