# Supplementary material for: 8-Amino-6-Methoxyquinoline—Tetrazole Hybrids: Impact of Linkers on Antiplasmodial Activity
Source: Molecules. 2021 Sep 12;26(18):5530. doi: 10.3390/molecules26185530 (PMC8470823; doi:10.3390/molecules26185530)

Supplementary Information

8-Amino-6-methoxyquinoline – Tetrazole hybrids: Impact of linkers on Antiplasmodial Activity

Patrick Hochegger [1,*], Johanna Dolensky [1], Werner Seebacher [1], Robert Saf [2], Marcel Kaiser [3], Pascal Mäser [3] and Robert Weis [1]

[1] Institute of Pharmaceutical Sciences, Pharmaceutical Chemistry, University of Graz, Schubertstraße 1, A-8010 Graz, Austria; theresa.hermann@uni-graz.at; patrick.hochegger@uni-graz.at; we.seebacher@uni-graz.at; robert.weis@uni-graz.at
[2] Institute for Chemistry and Technology of Materials (ICTM), Graz University of Technology, Stremayrgasse 9, A-8010 Graz, Austria; robert.saf@tugraz.at
[3] Swiss Tropical and Public Health Institute, Socinstraße 57, CH-4002 Basel, Switzerland; marcel.kaiser@swisstph.ch; pascal.maeser@swisstph.ch
* Correspondence: patrick.hochegger@uni-graz.at; Tel.: +43-316-380-5379; fax: +43-316-380-9846

NMR spectra data of compounds 3–22

Figure S1. 1H NMR at 400 MHz and 13C NMR at 100 MHz spectra for compound **3**

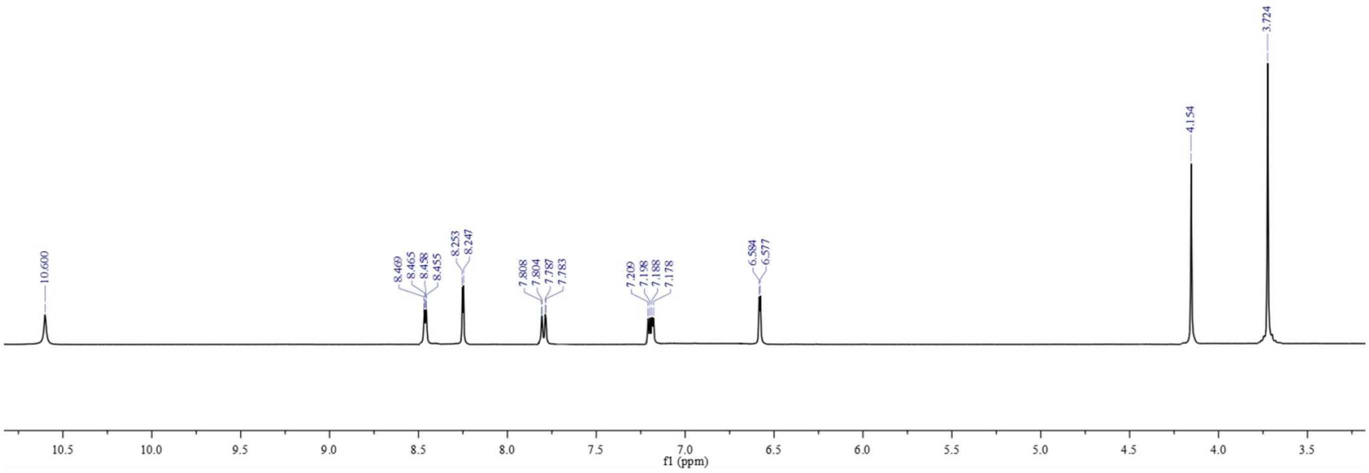

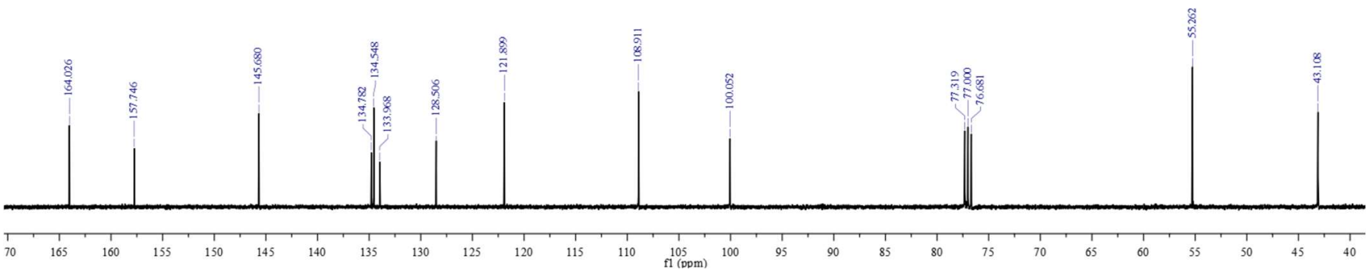

Figure S2. 1H NMR at 400 MHz and 13C NMR at 100 MHz spectra for compound **4**

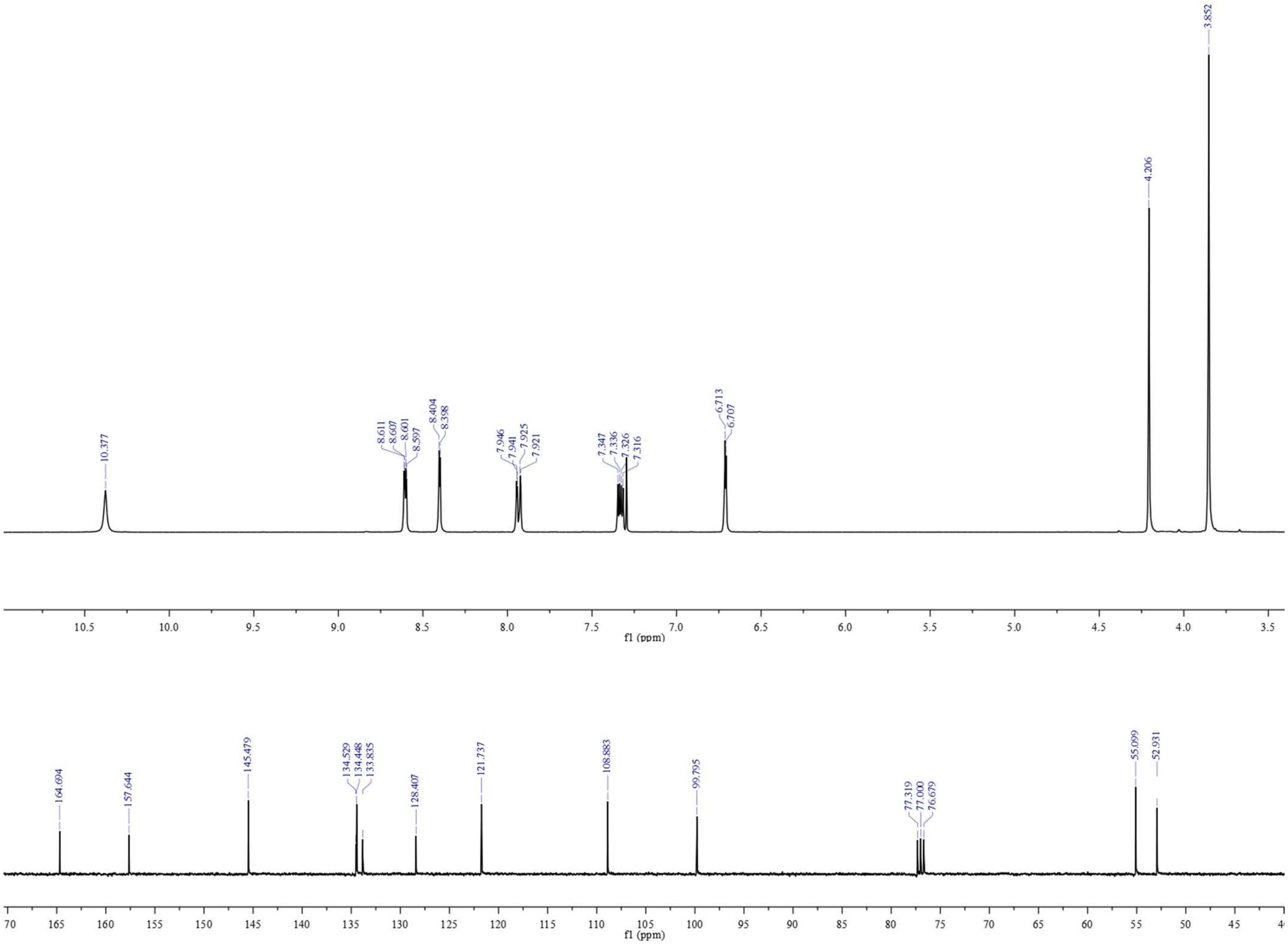

Figure S3. 1H NMR at 400 MHz and 13C NMR at 100 MHz spectra for compound **5**

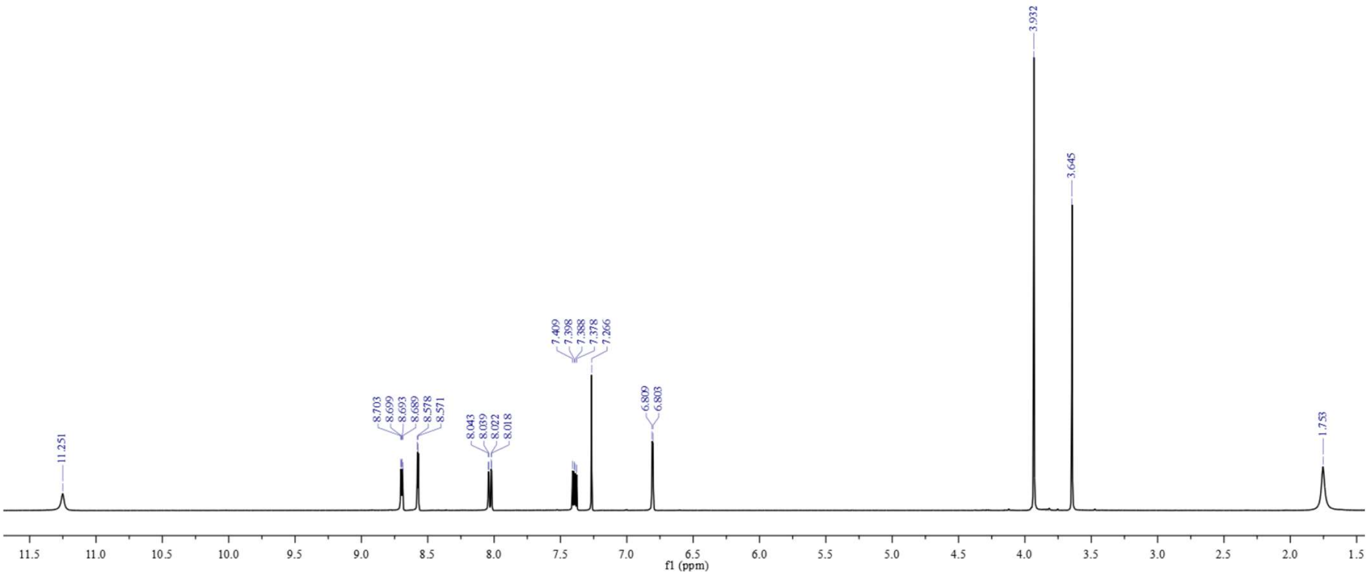

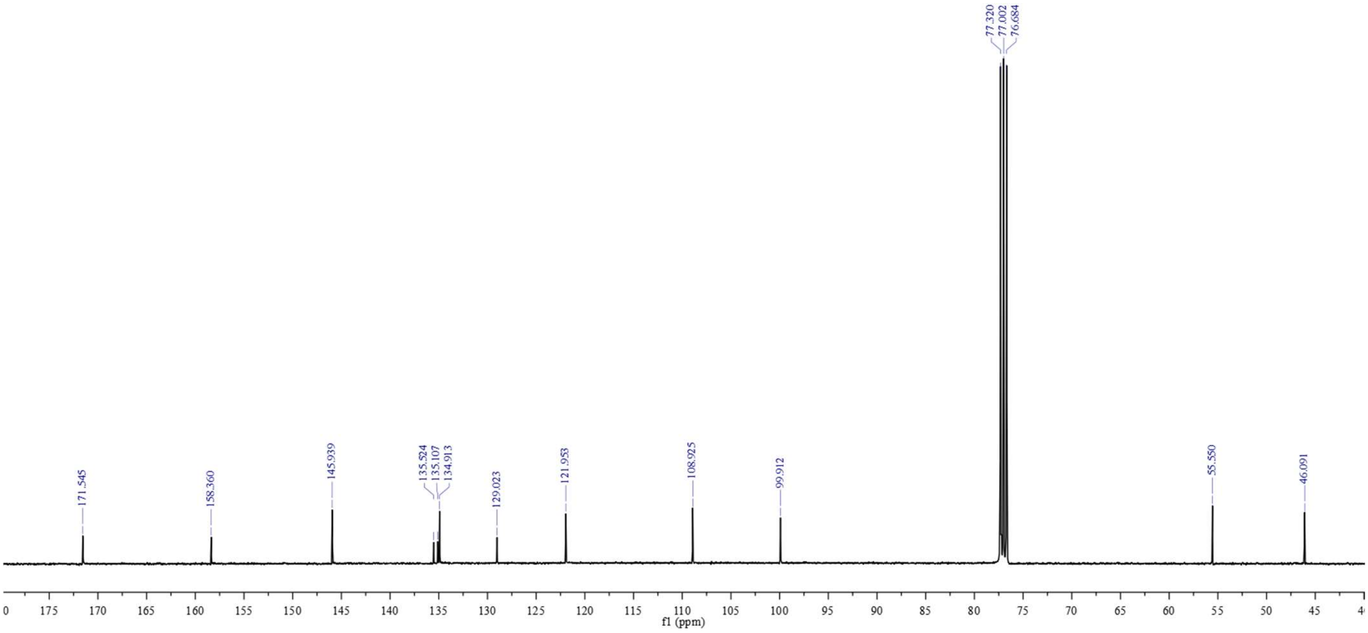

Figure S4. 1H NMR at 400 MHz and 13C NMR at 100 MHz spectra for compound **6**

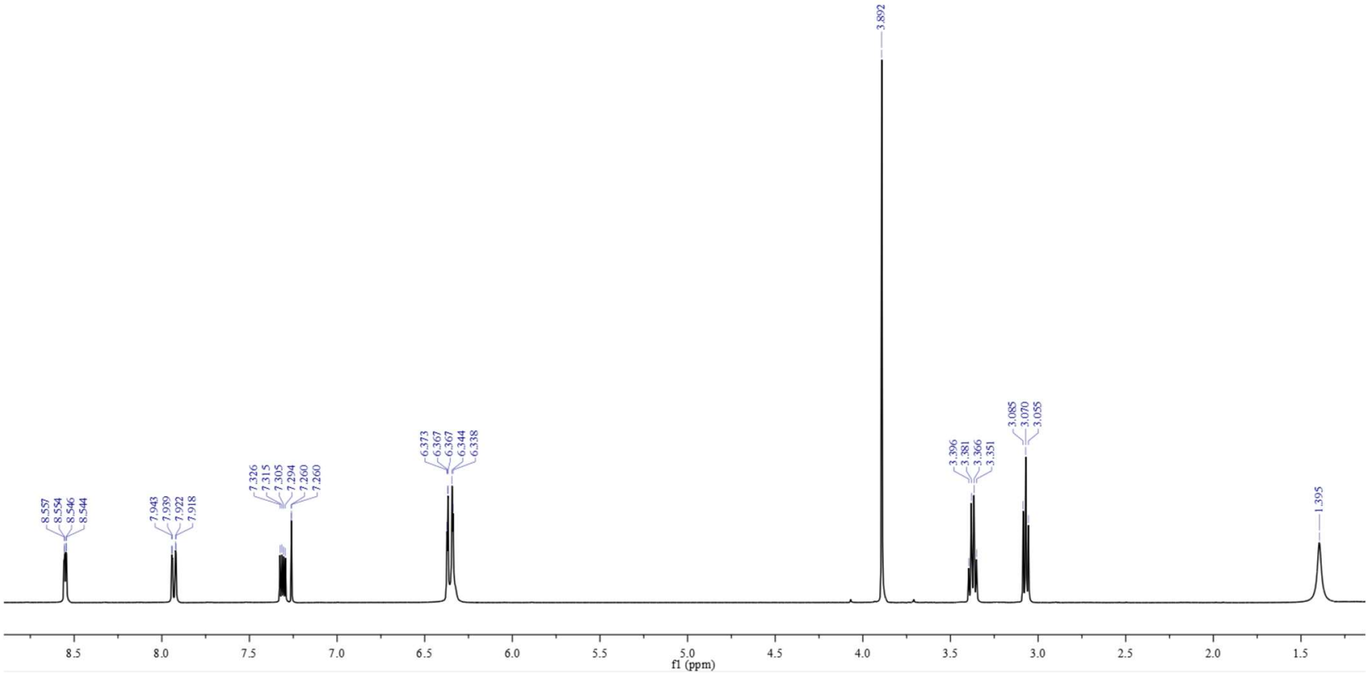

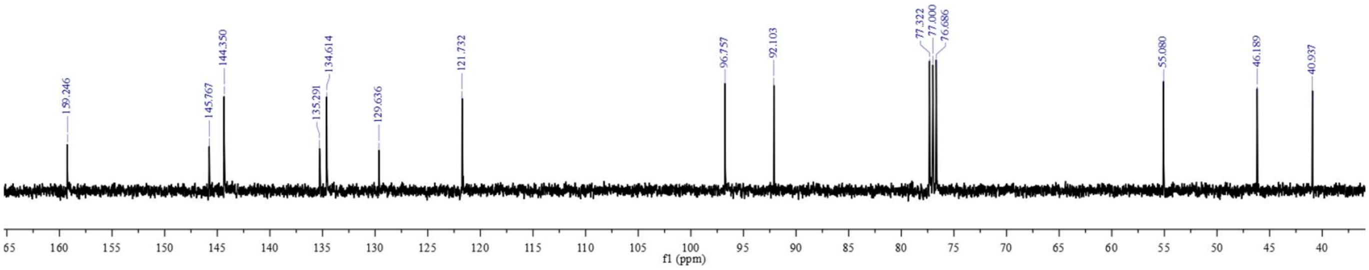

Figure S5. 1H NMR at 400 MHz and 13C NMR at 100 MHz spectra for compound **7**

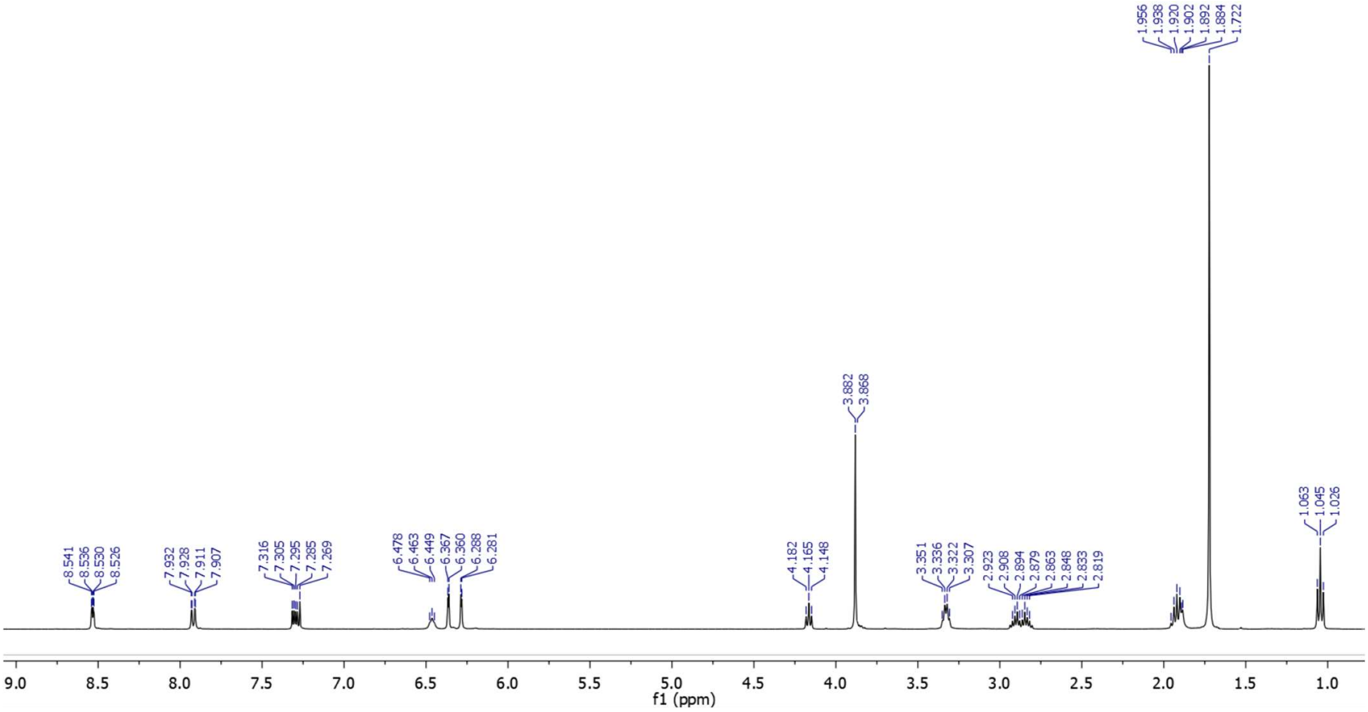

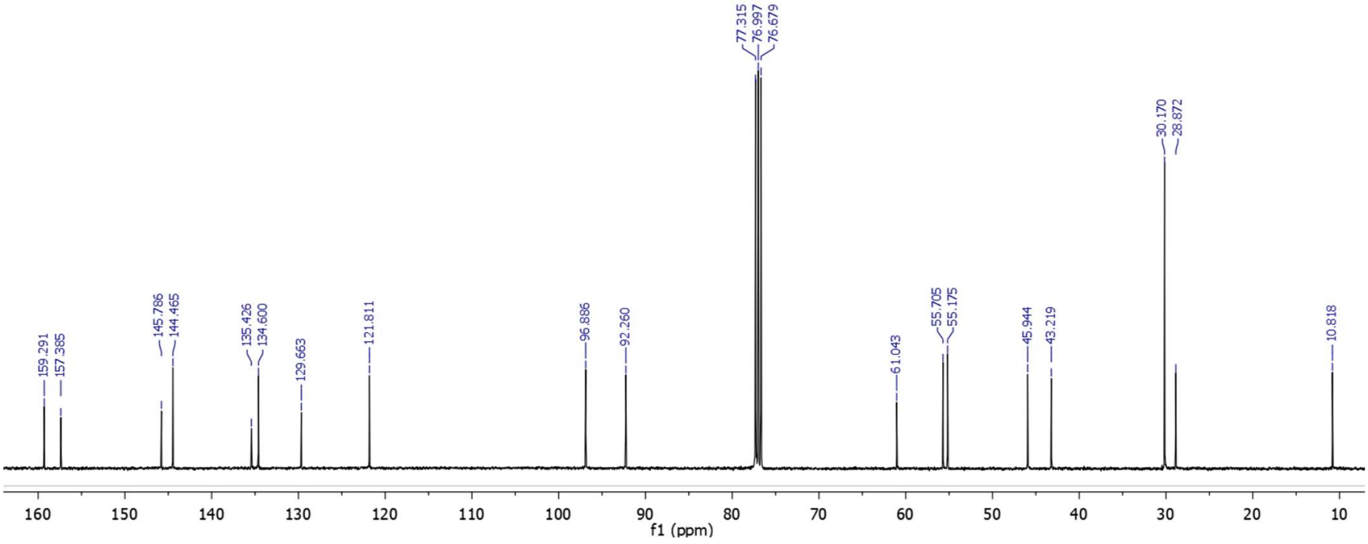

Figure S6. 1H NMR at 400 MHz and 13C NMR at 100 MHz spectra for compound **8**

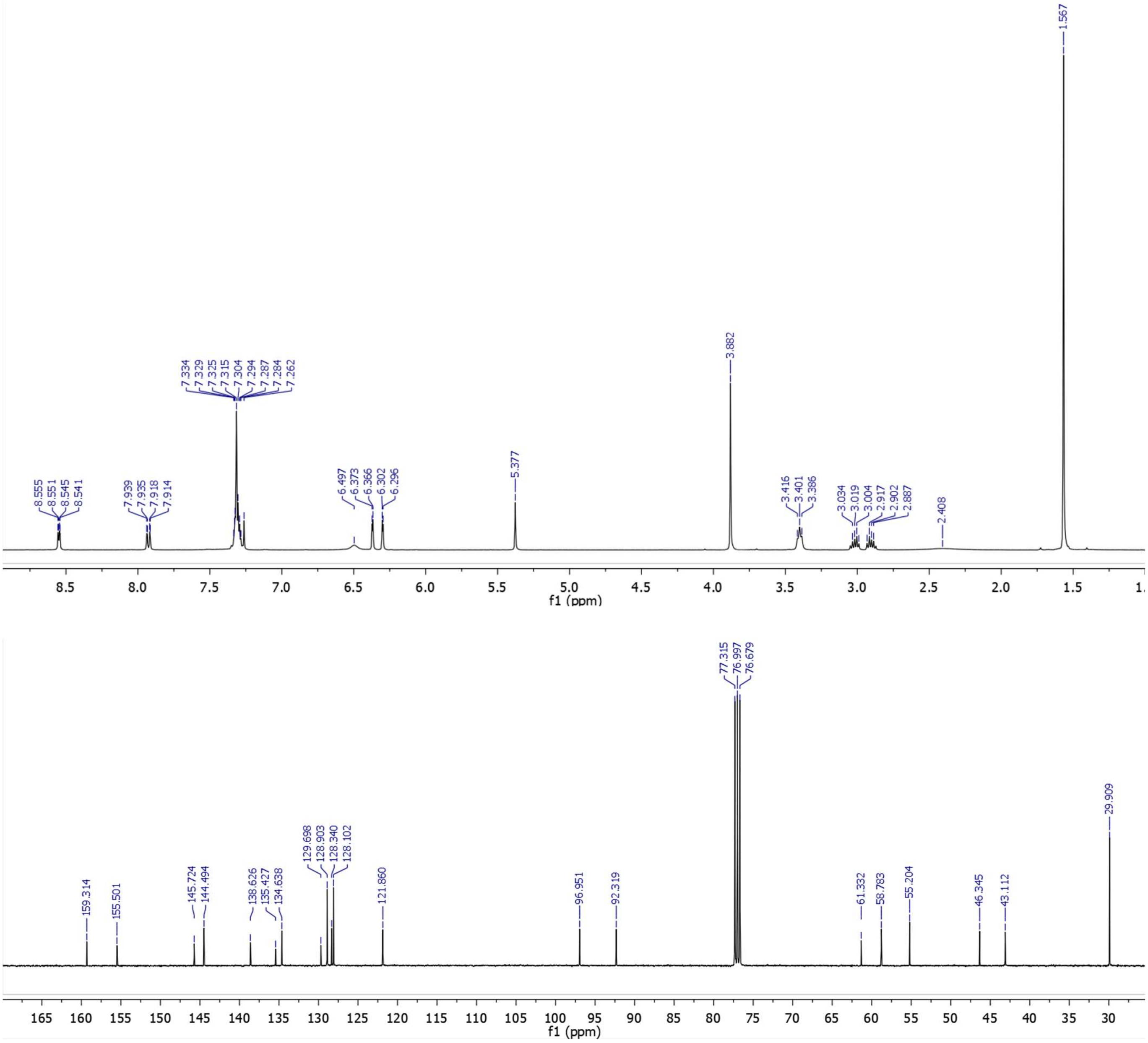

Figure S7. 1H NMR at 400 MHz and 13C NMR at 100 MHz spectra for compound **9**

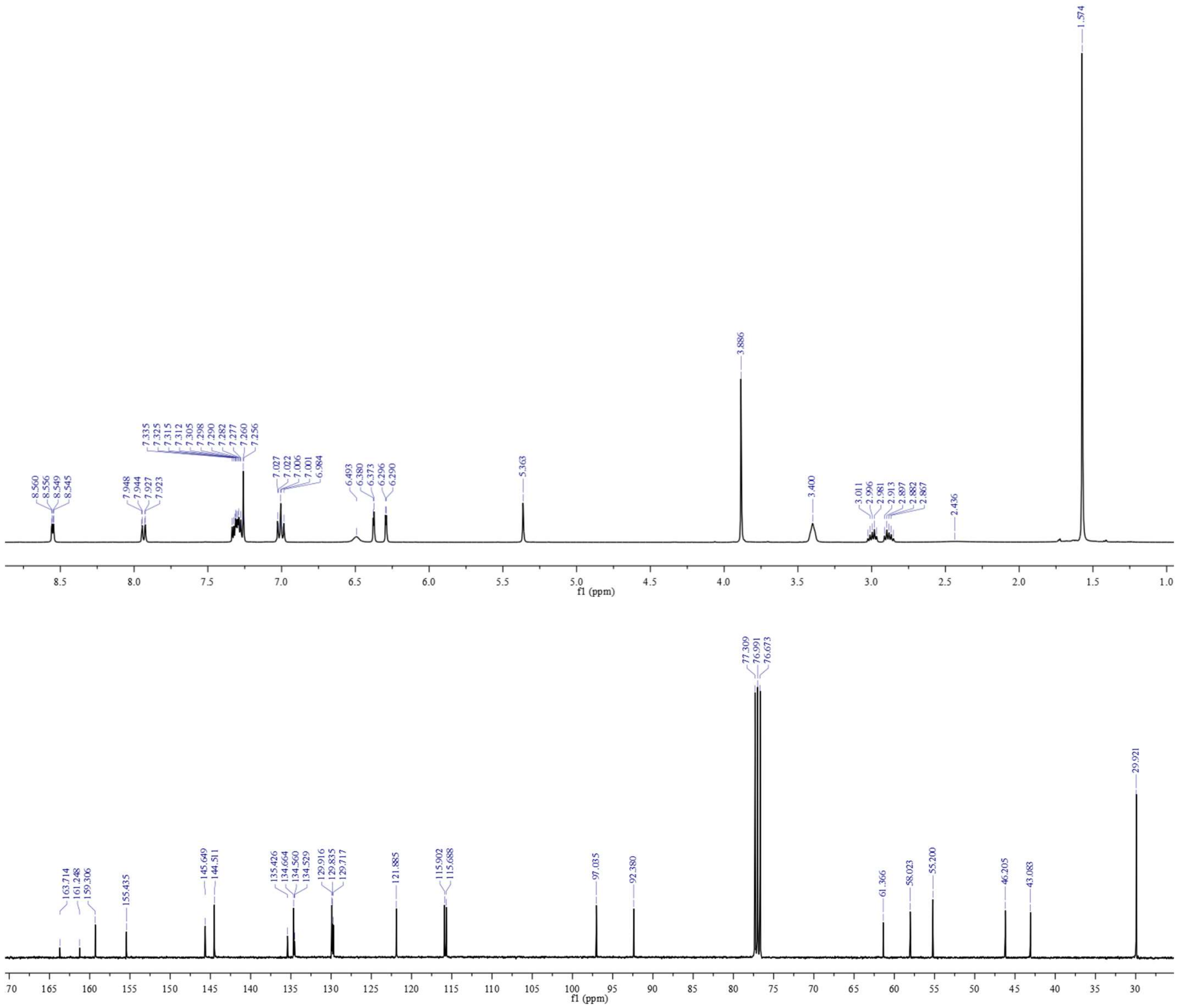

Figure S8. 1H NMR at 400 MHz and 13C NMR at 100 MHz spectra for compound **10**

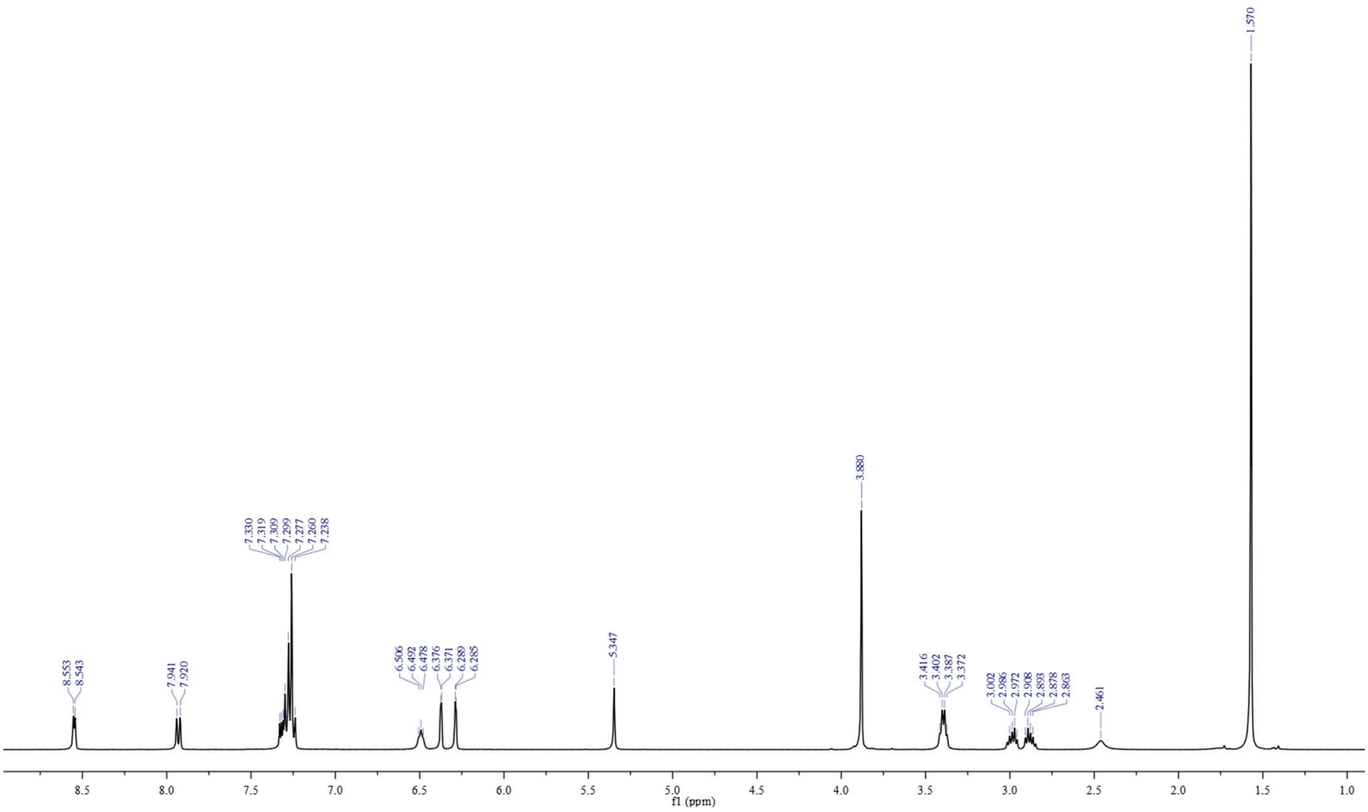

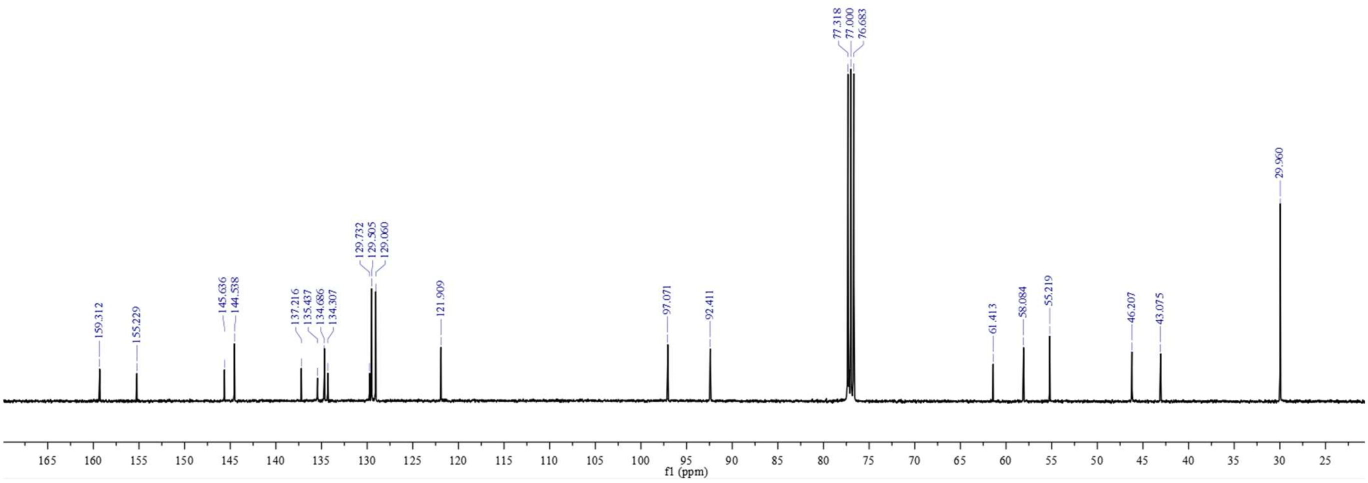

Figure S9. 1H NMR at 400 MHz and 13C NMR at 100 MHz spectra for compound **11**

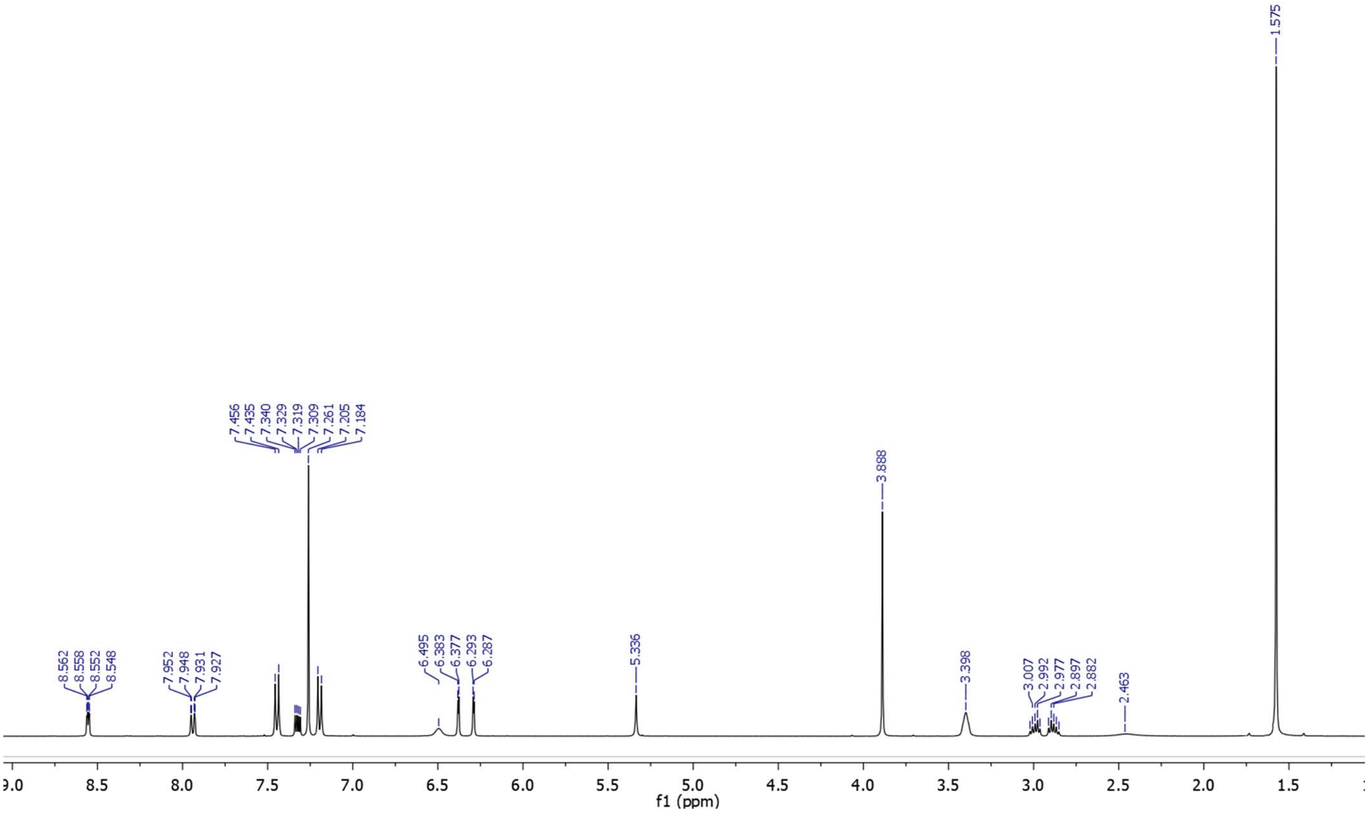

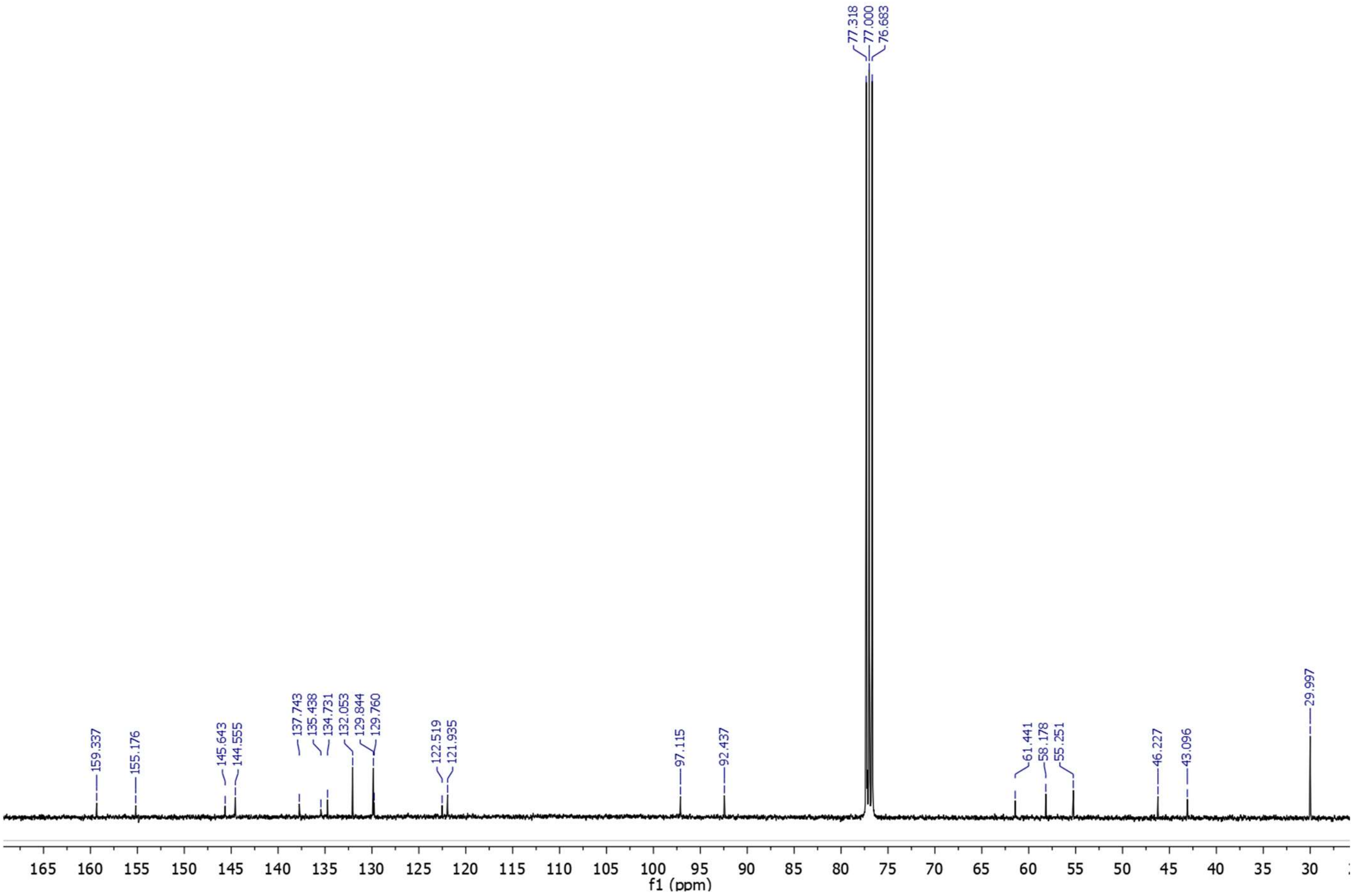

Figure S10. 1H NMR at 400 MHz and 13C NMR at 100 MHz spectra for compound **12**

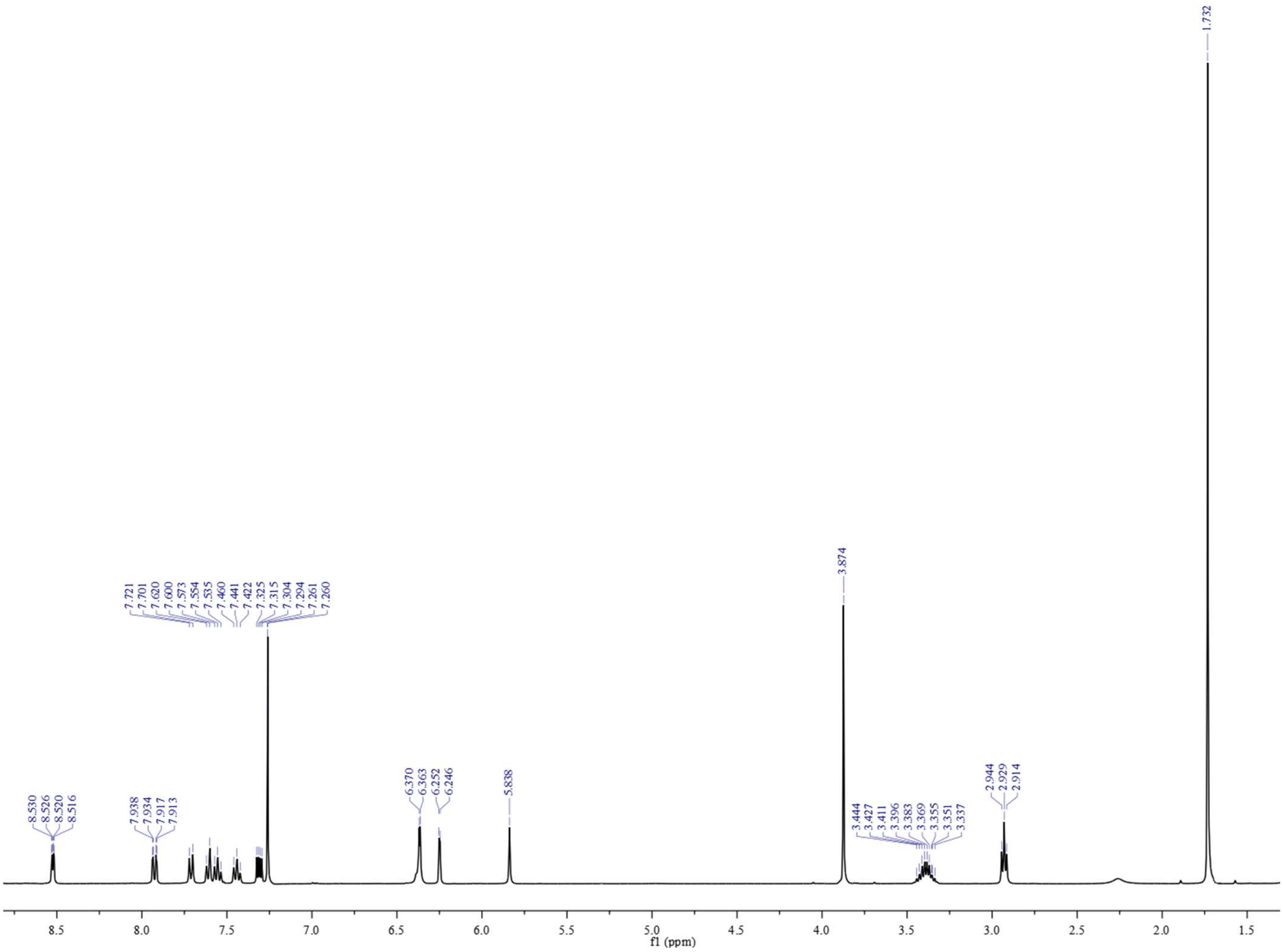

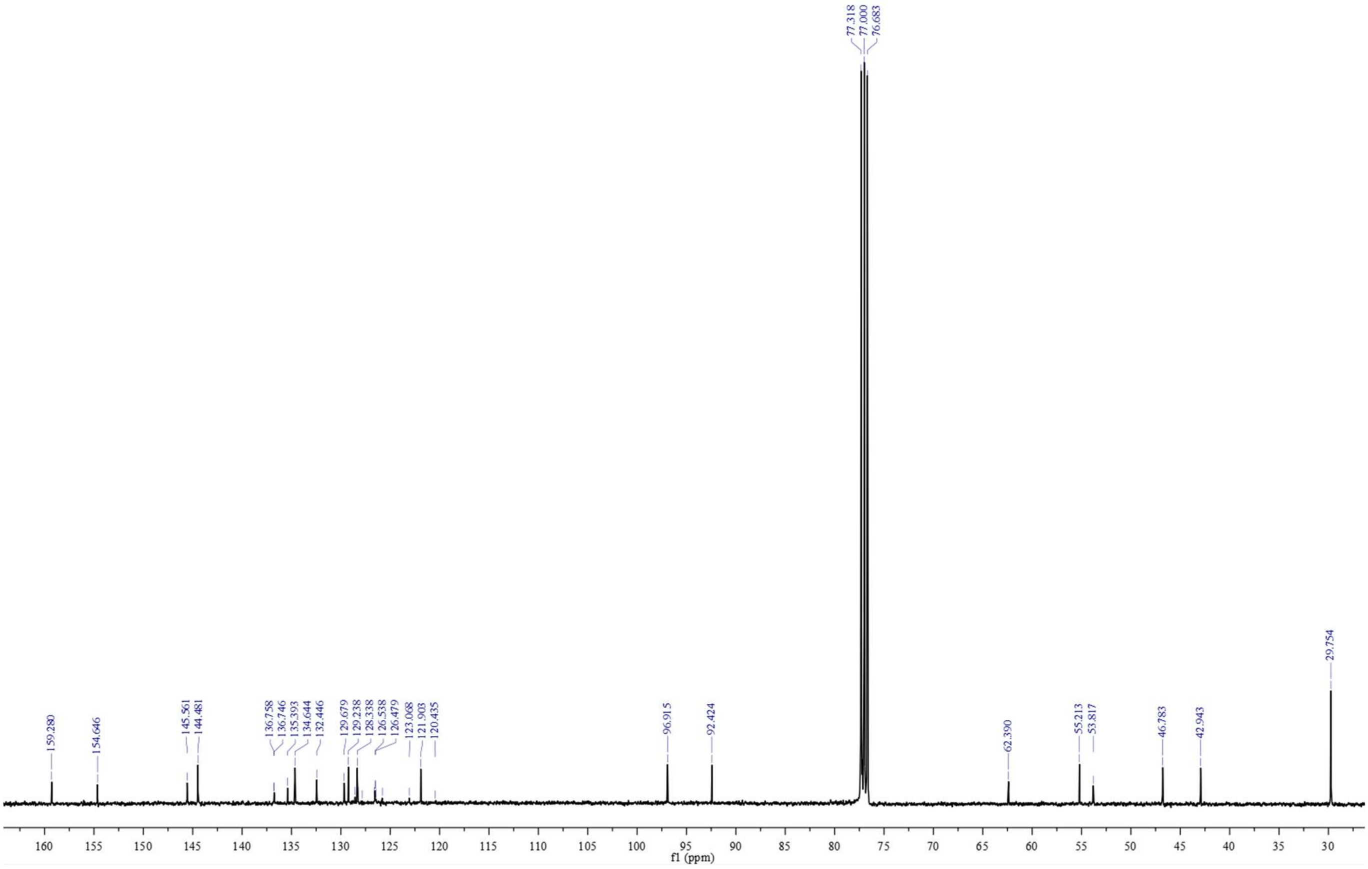

Figure S11. 1H NMR at 400 MHz and 13C NMR at 100 MHz spectra for compound **13**

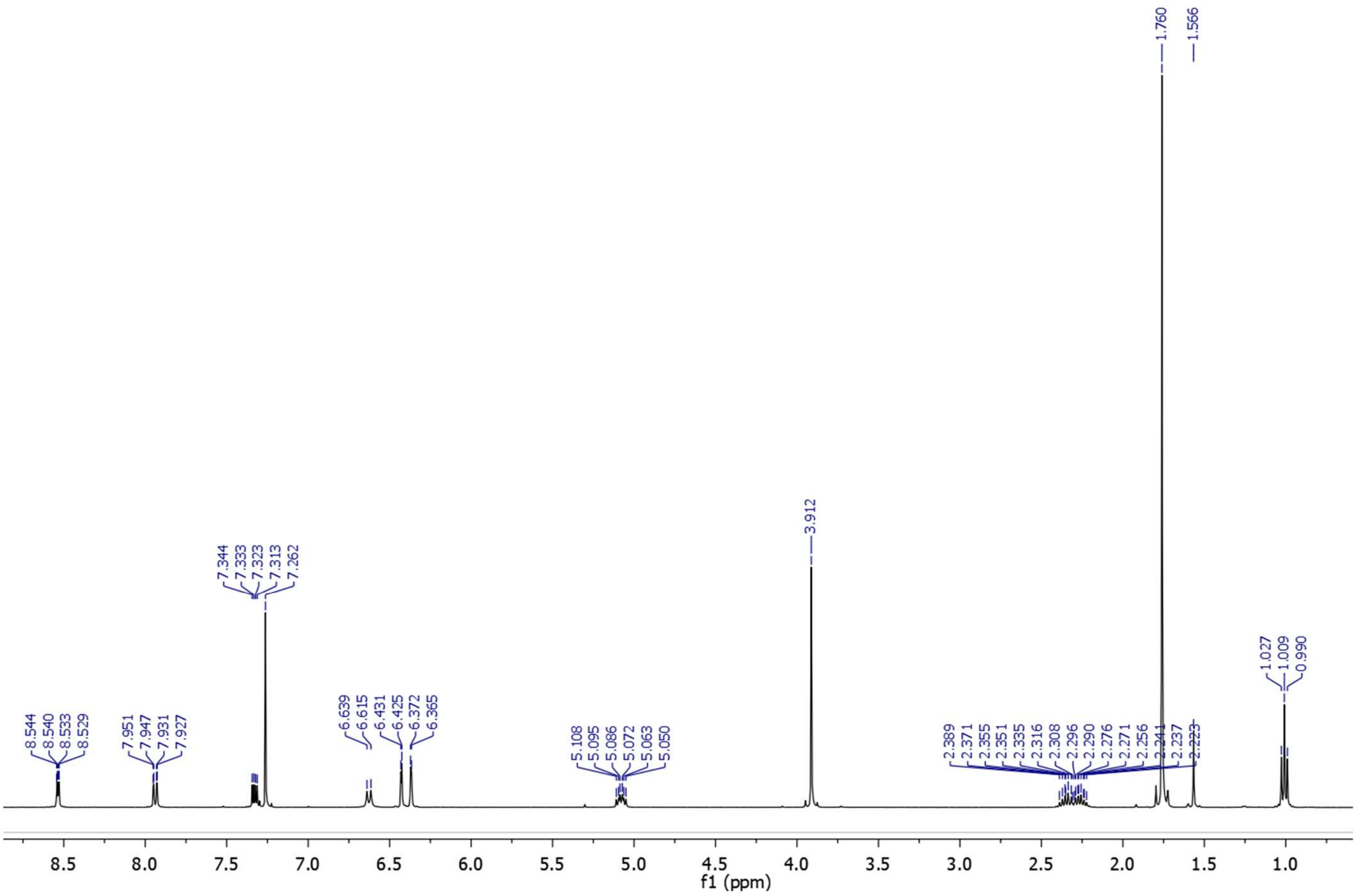

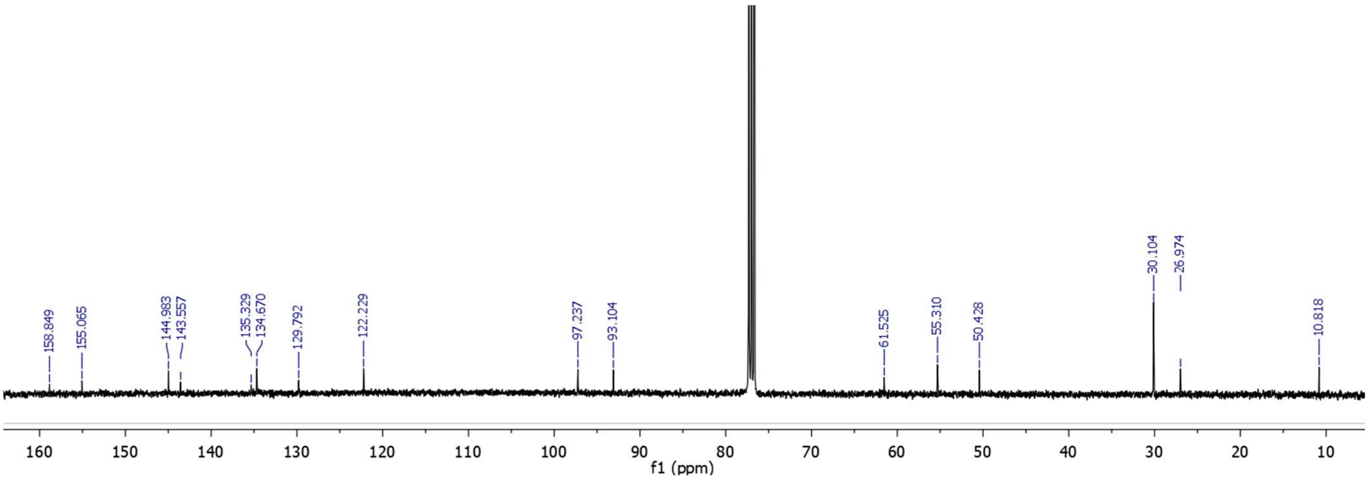

Figure S12. 1H NMR at 400 MHz and 13C NMR at 100 MHz spectra for compound **14**

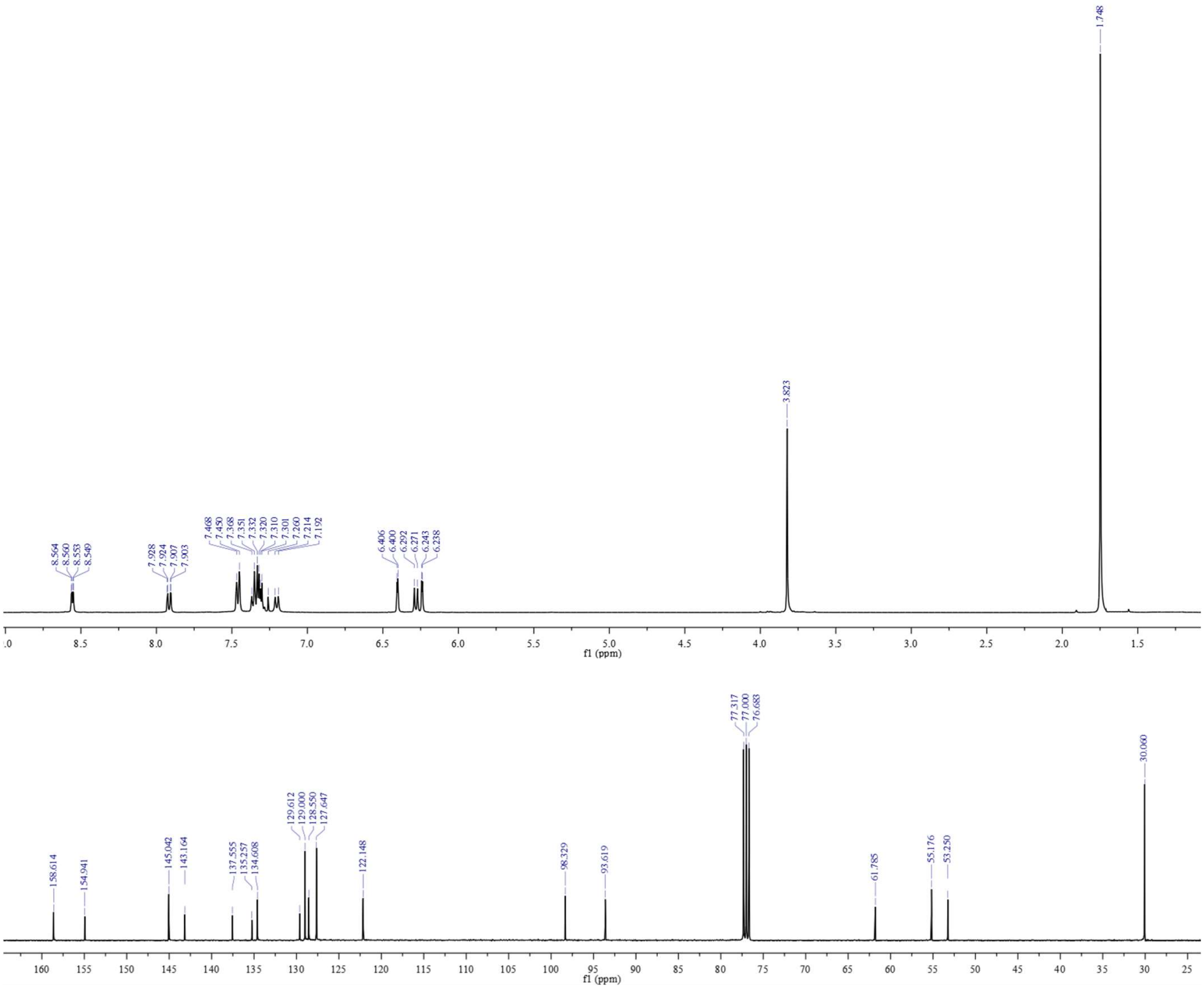

Figure S13. 1H NMR at 400 MHz and 13C NMR at 100 MHz spectra for compound **15**

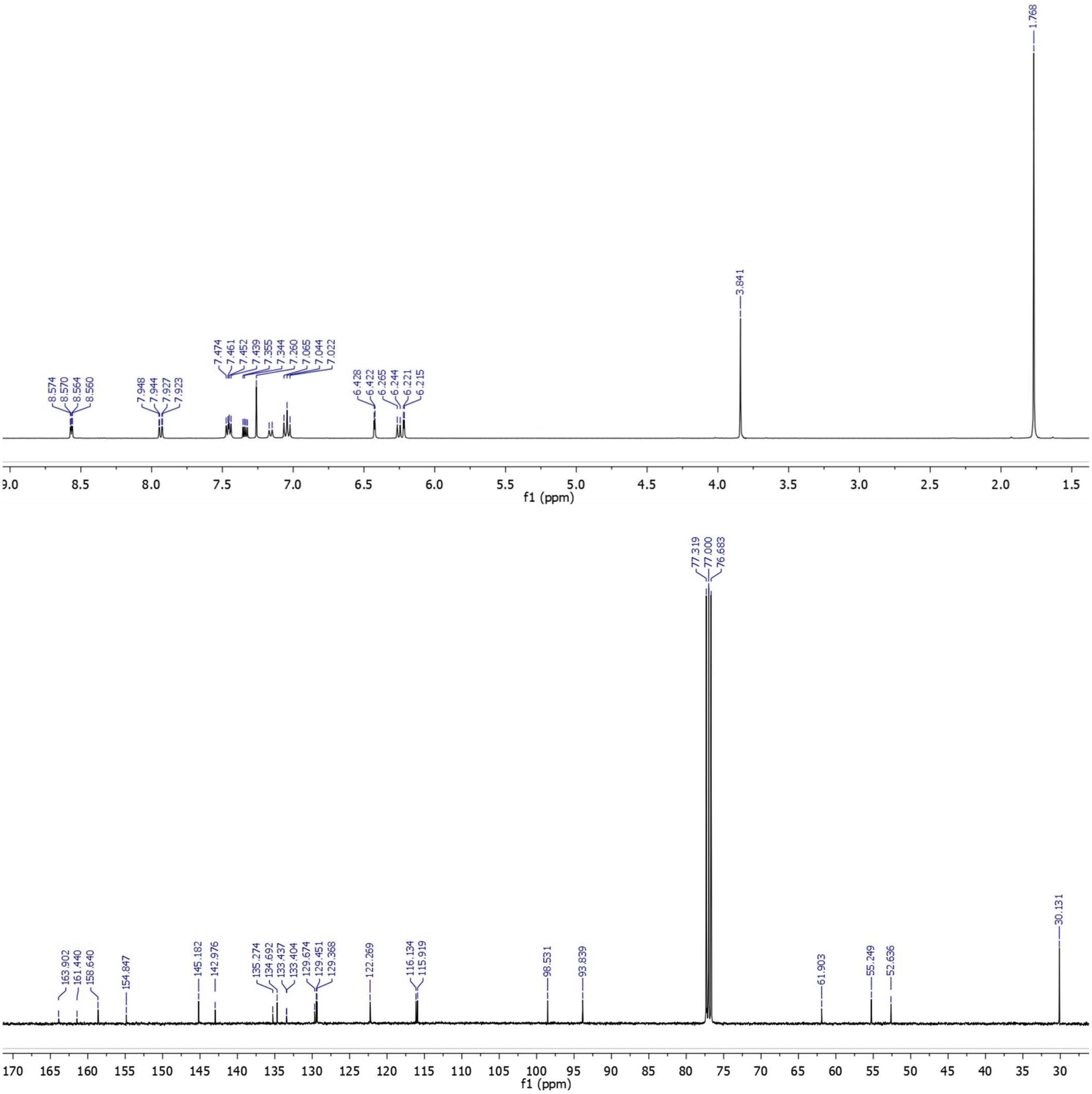

Figure S14. 1H NMR at 400 MHz and 13C NMR at 100 MHz spectra for compound **16**

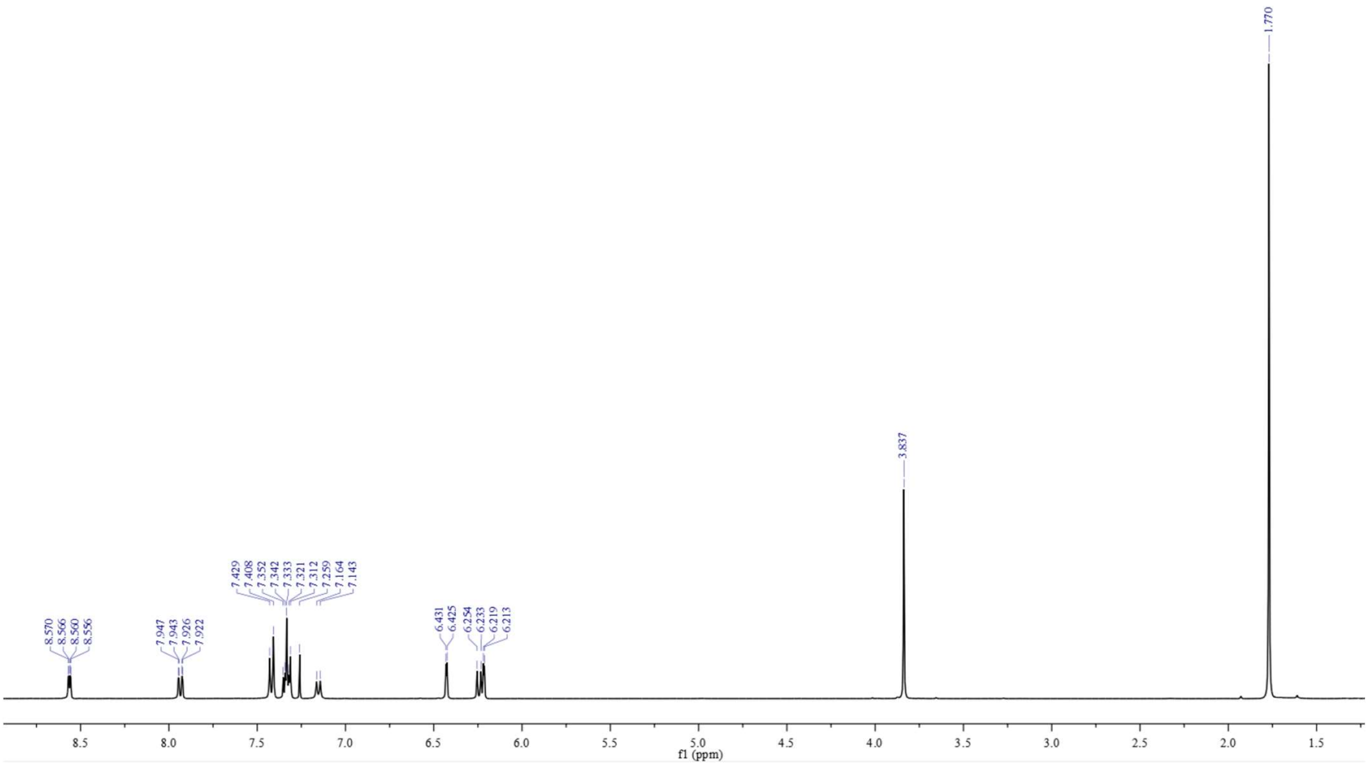

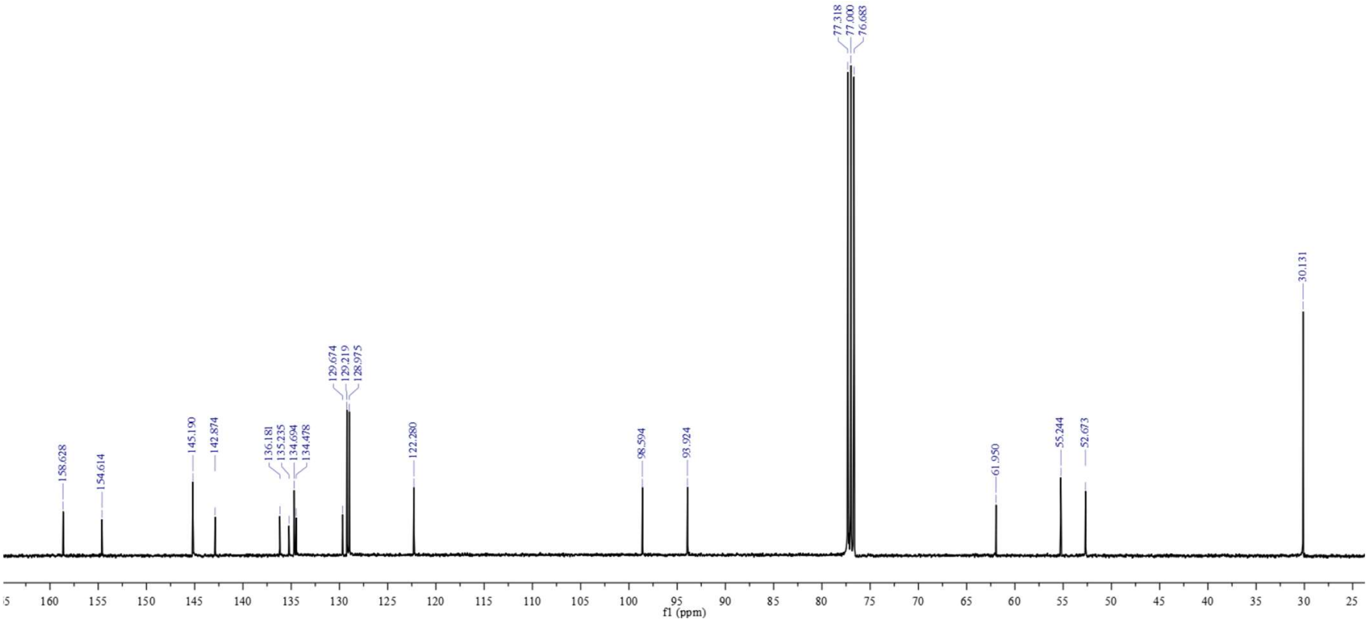

Figure S15. 1H NMR at 400 MHz and 13C NMR at 100 MHz spectra for compound **17**

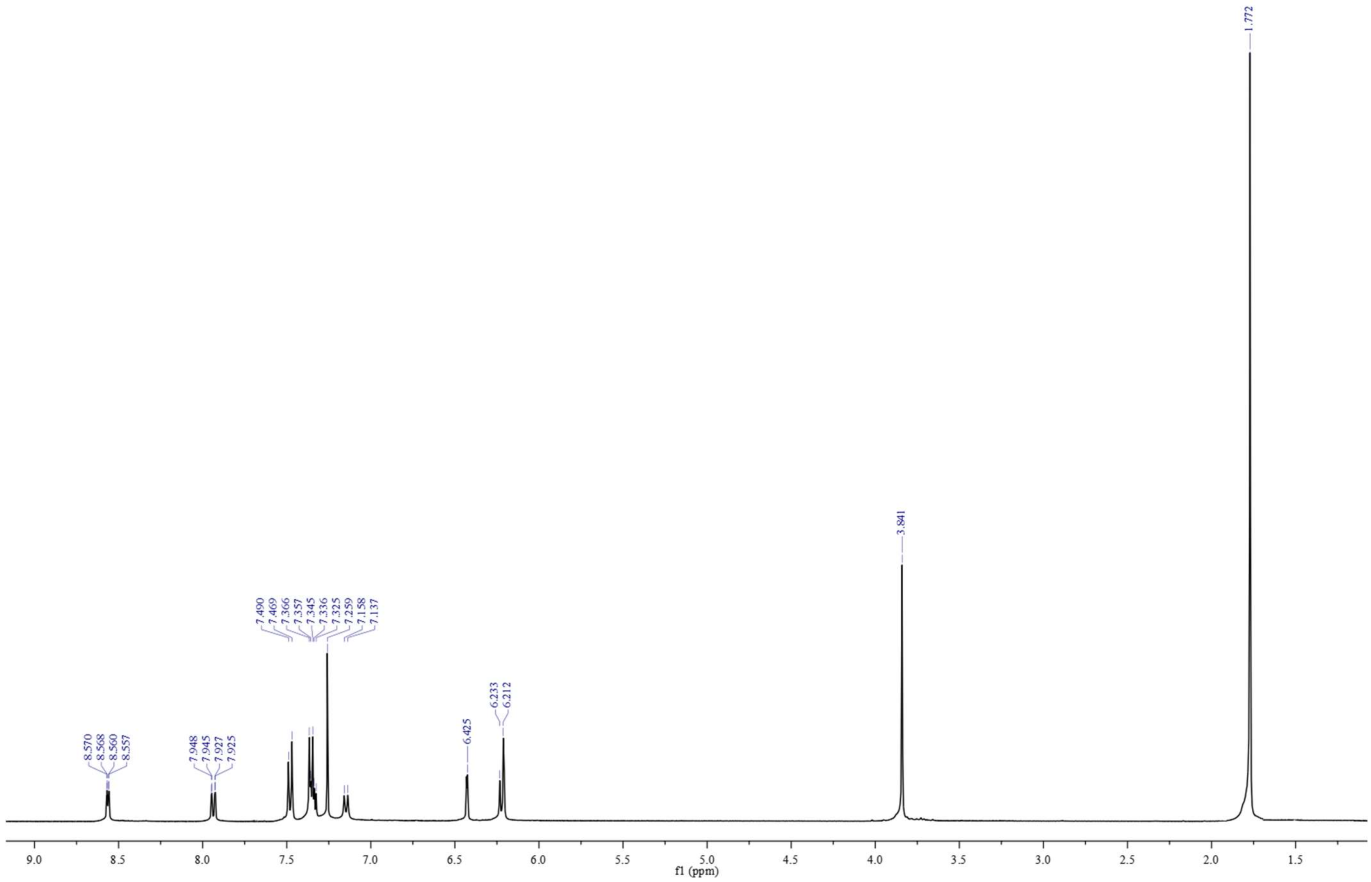

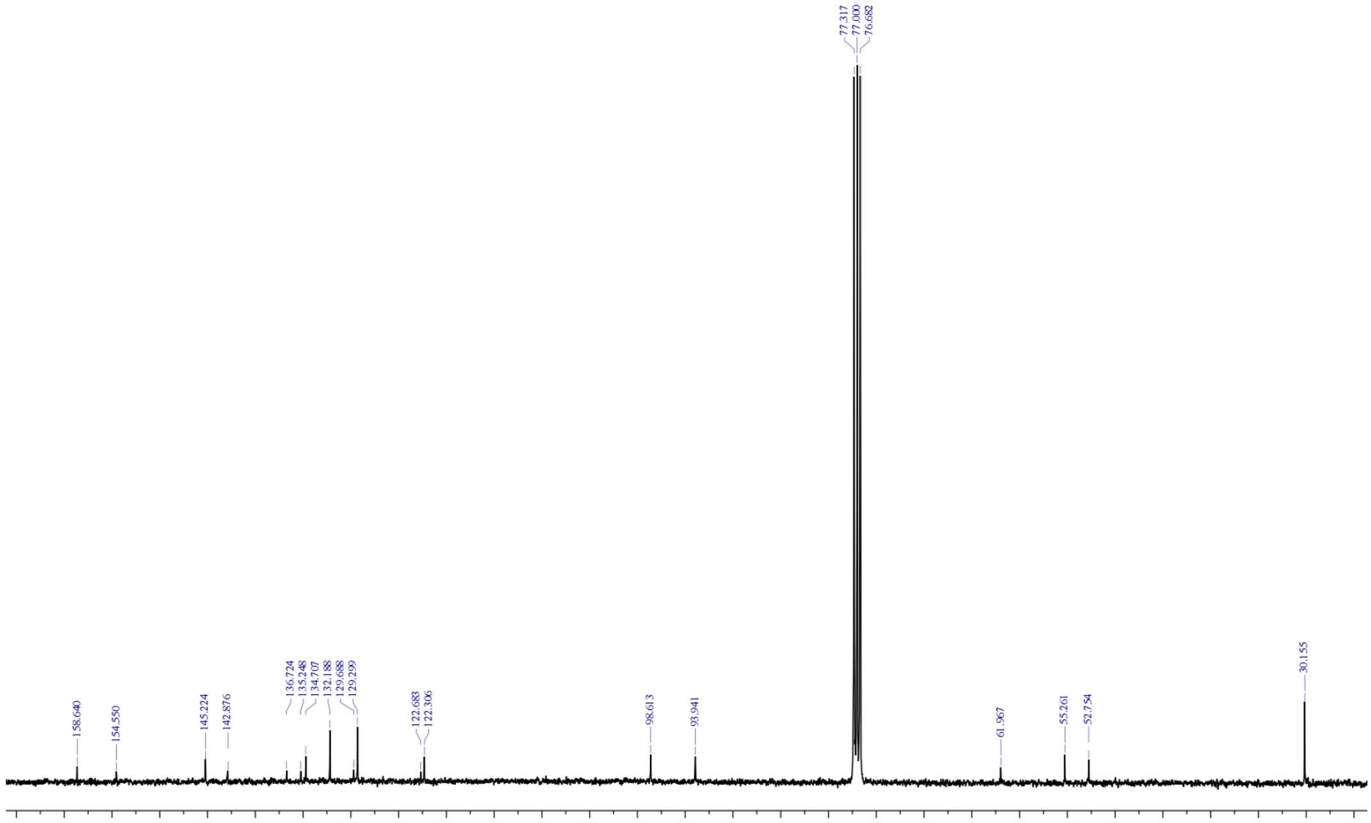

Figure S16. 1H NMR at 400 MHz and 13C NMR at 100 MHz spectra for compound **18**

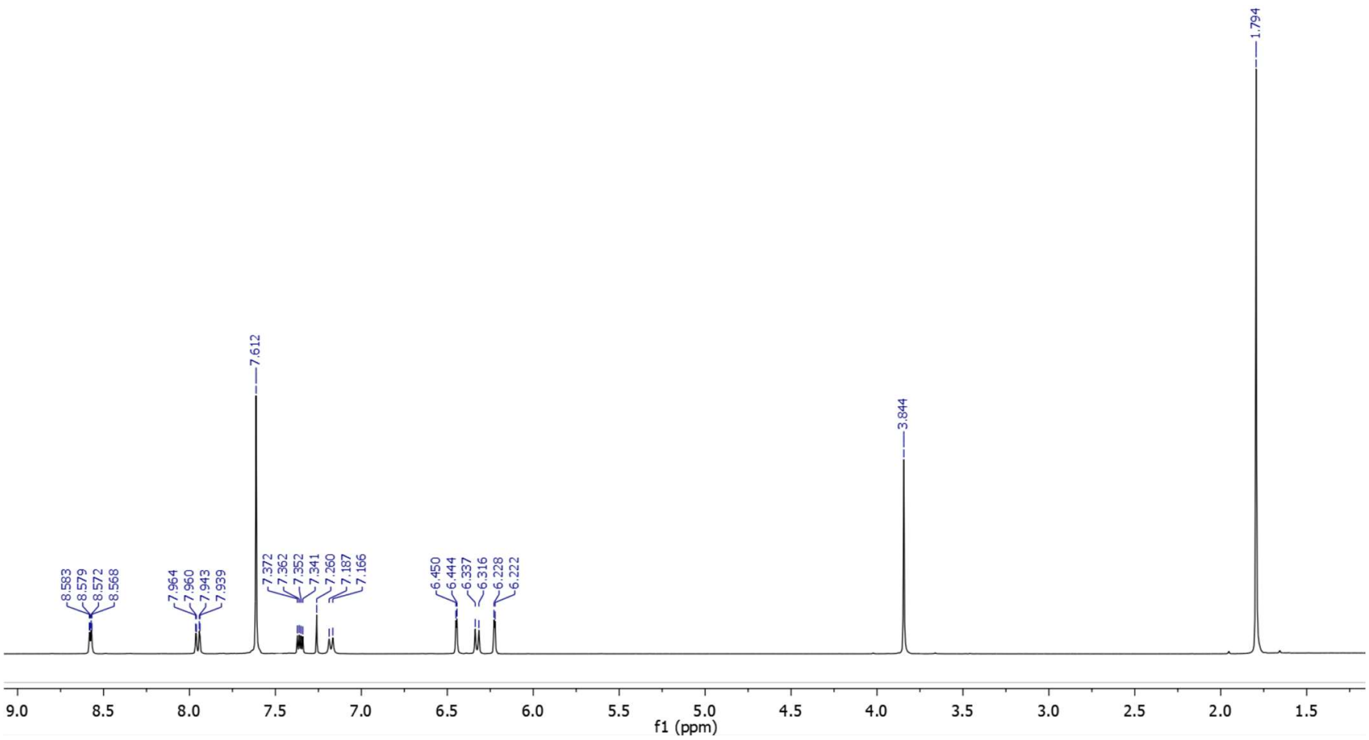

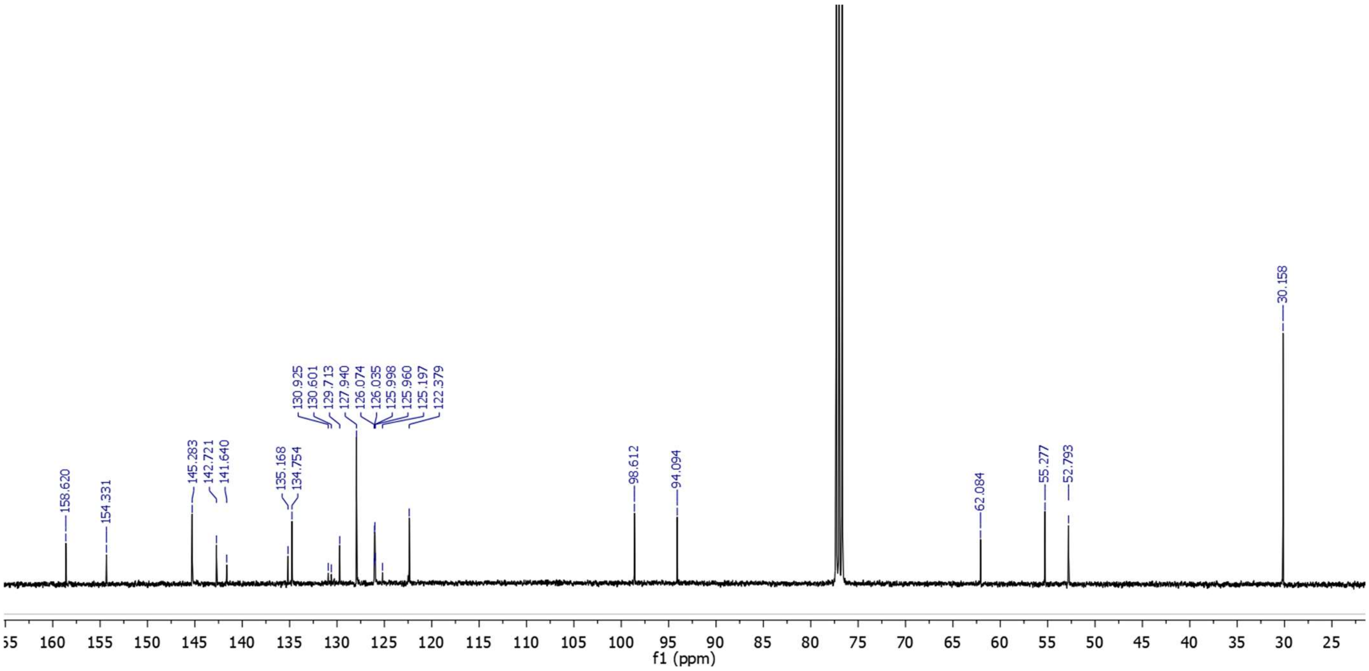

Figure S17. 1H NMR at 400 MHz and 13C NMR at 100 MHz spectra for compound **19**

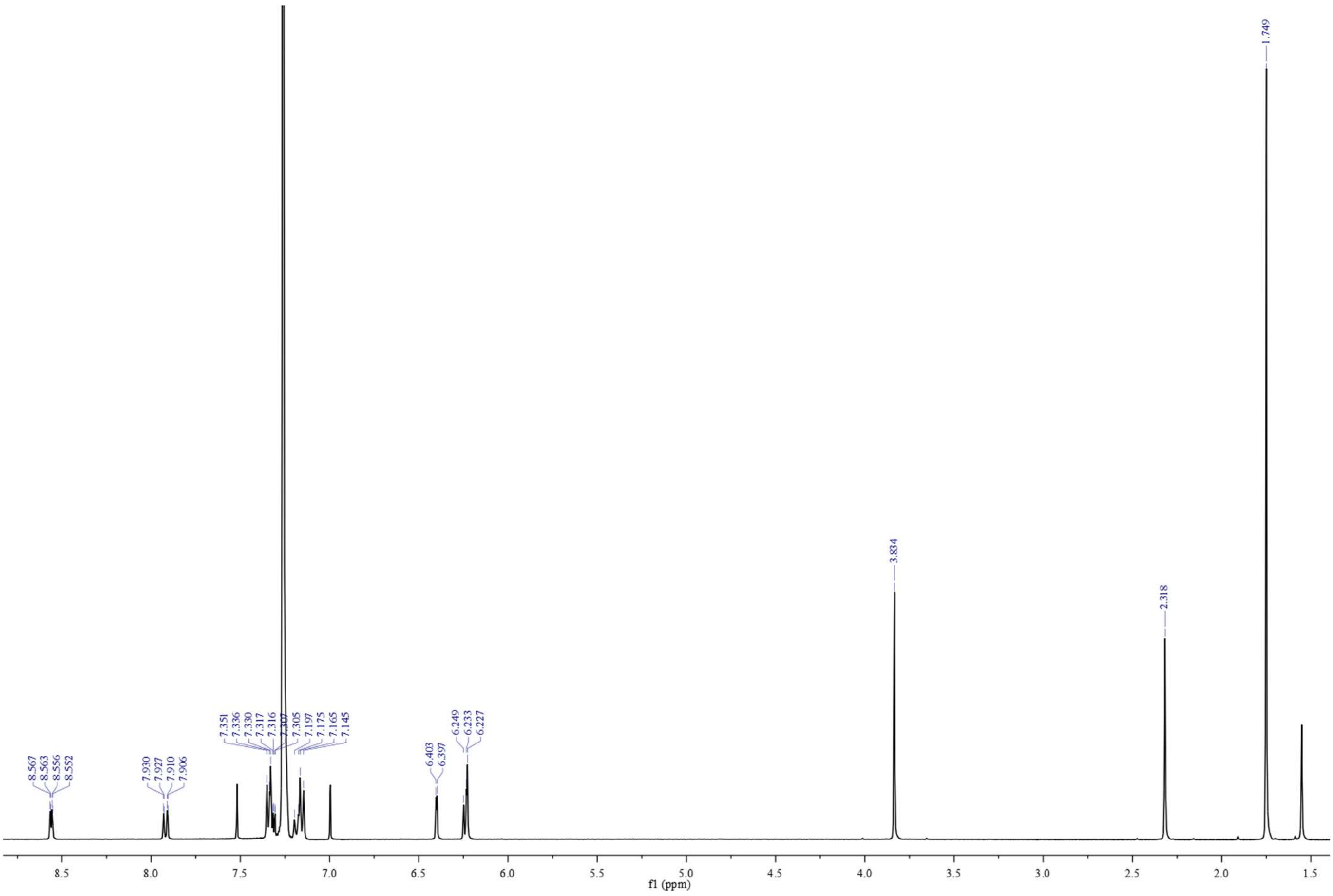

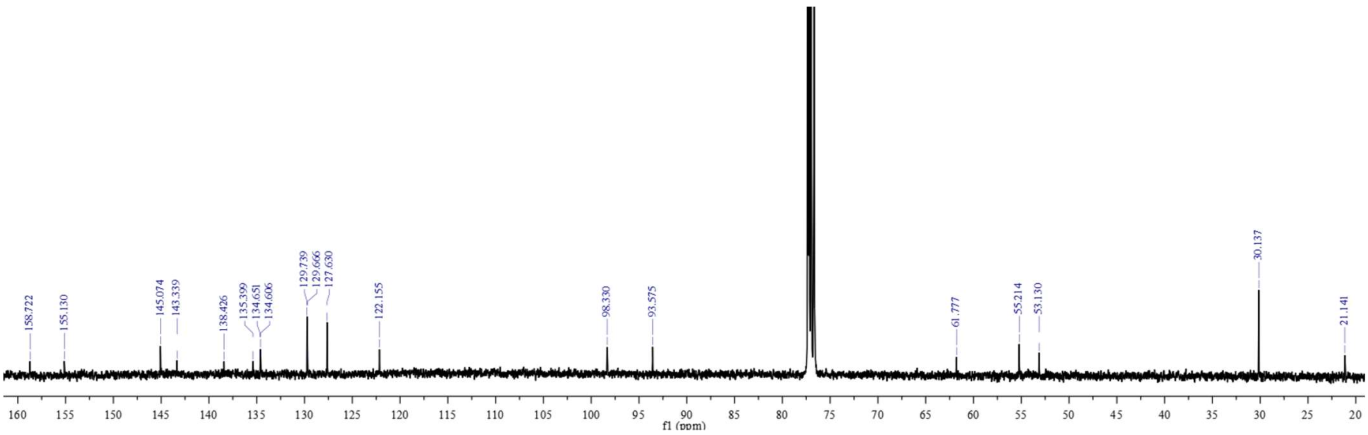

Figure S18. 1H NMR at 400 MHz and 13C NMR at 100 MHz spectra for compound **20**

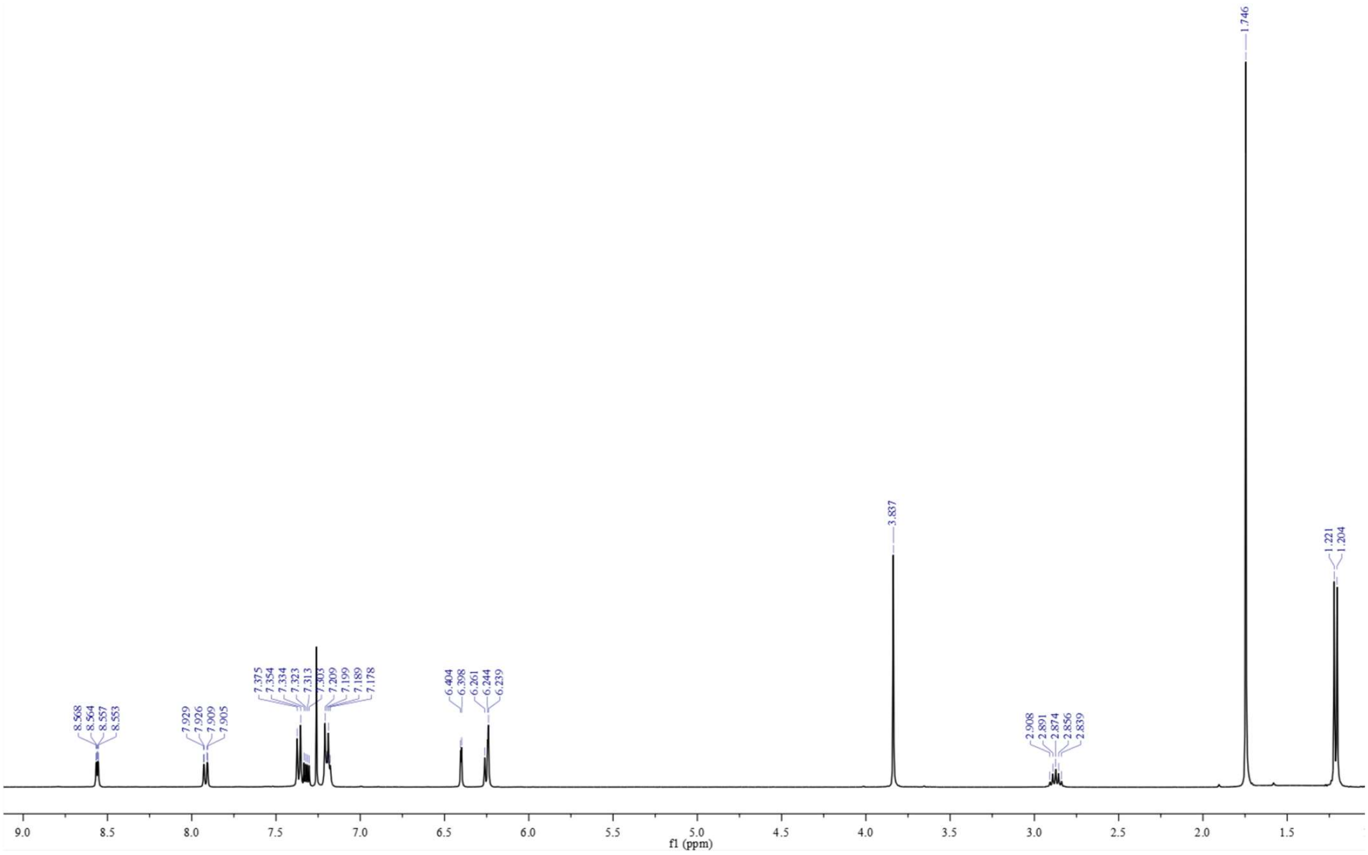

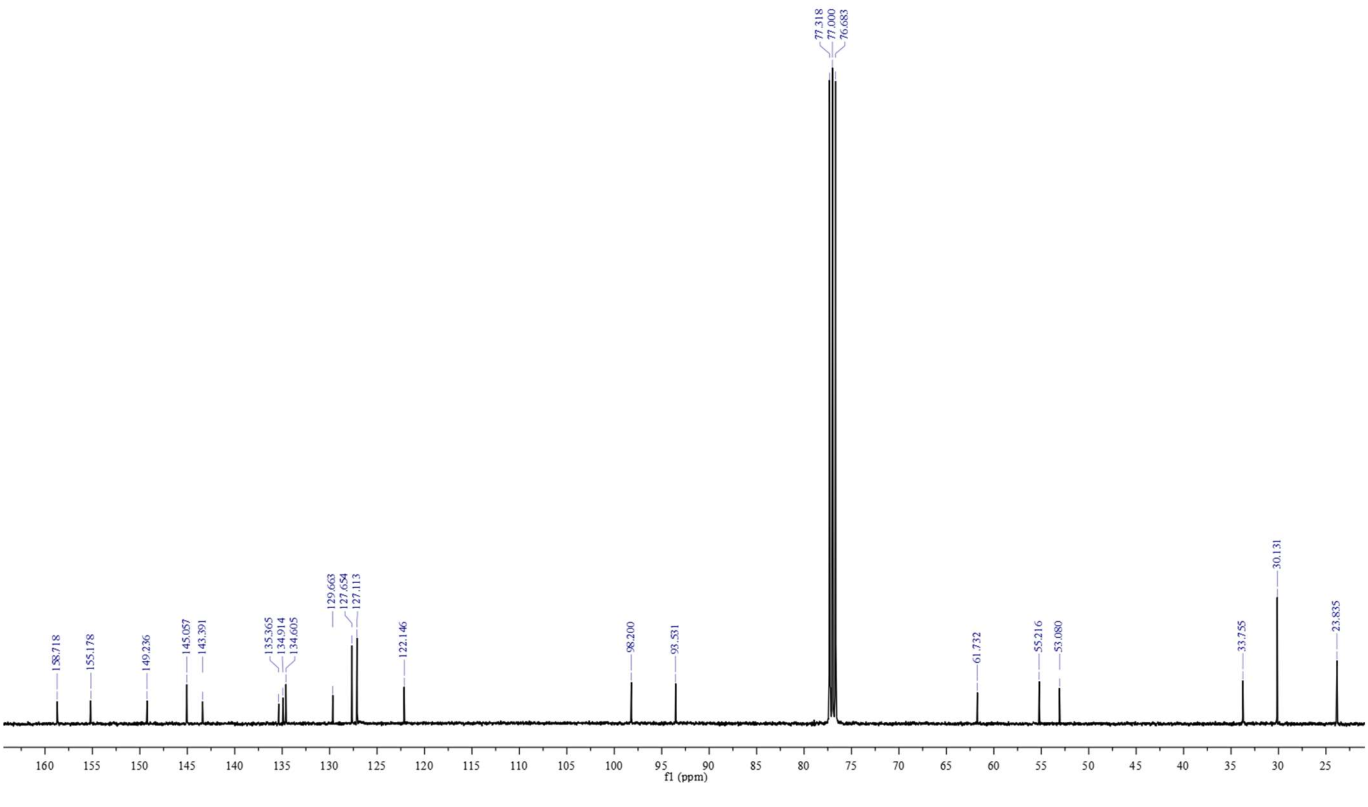

Figure S19. 1H NMR at 400 MHz and 13C NMR at 100 MHz spectra for compound **21**

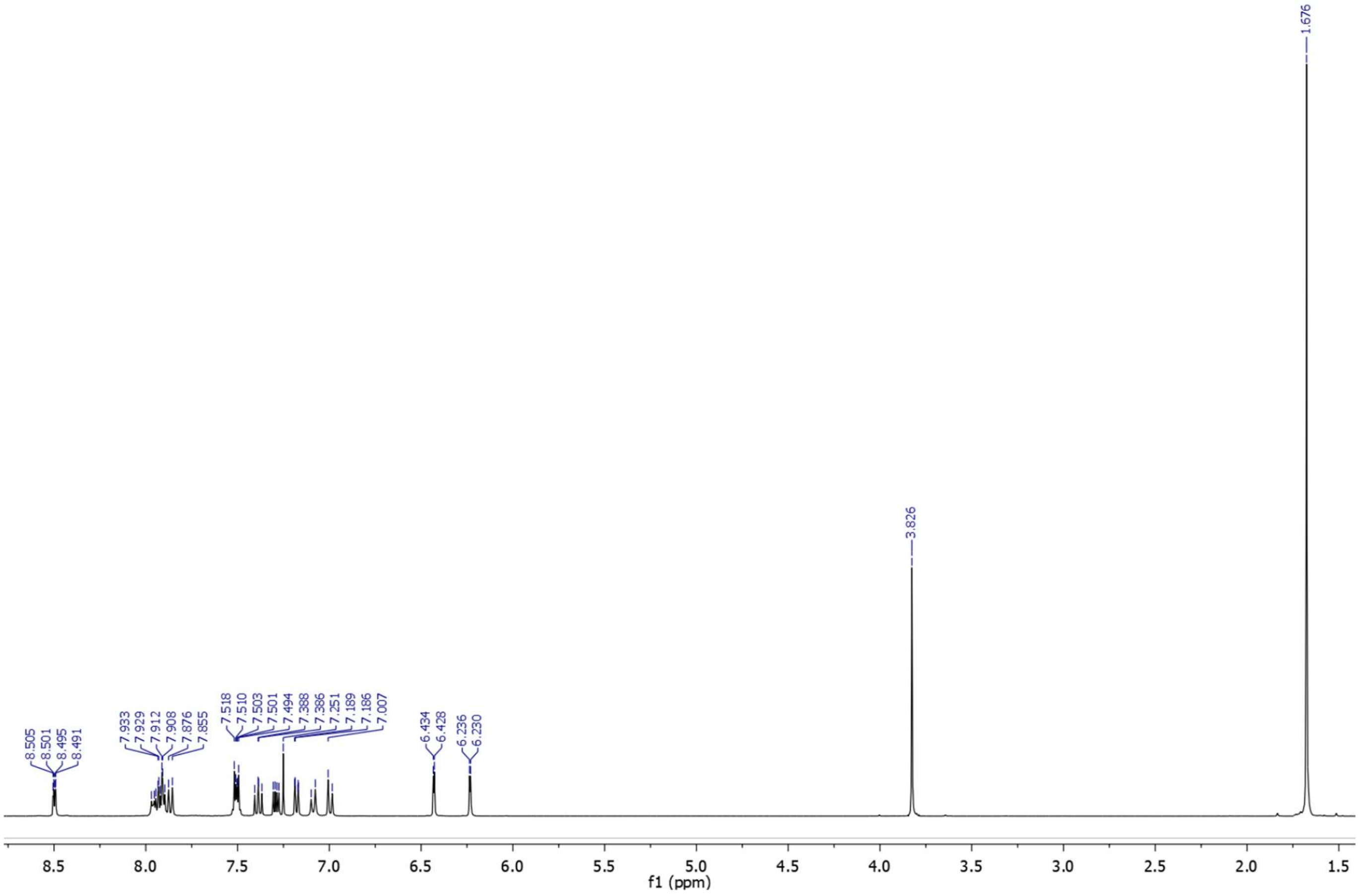

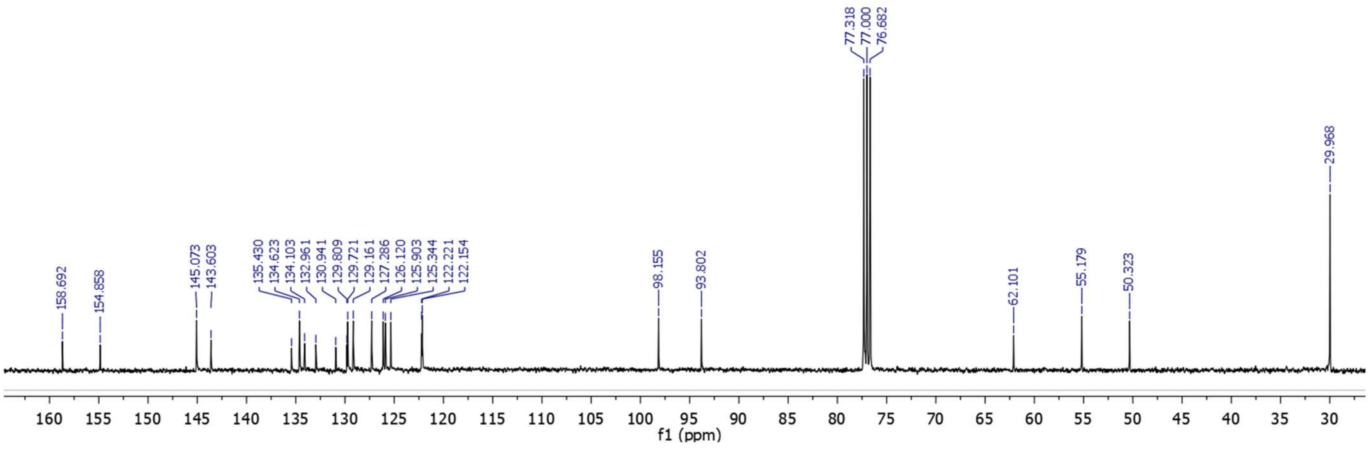

Figure S20. 1H NMR at 400 MHz and 13C NMR at 100 MHz spectra for compound **22**

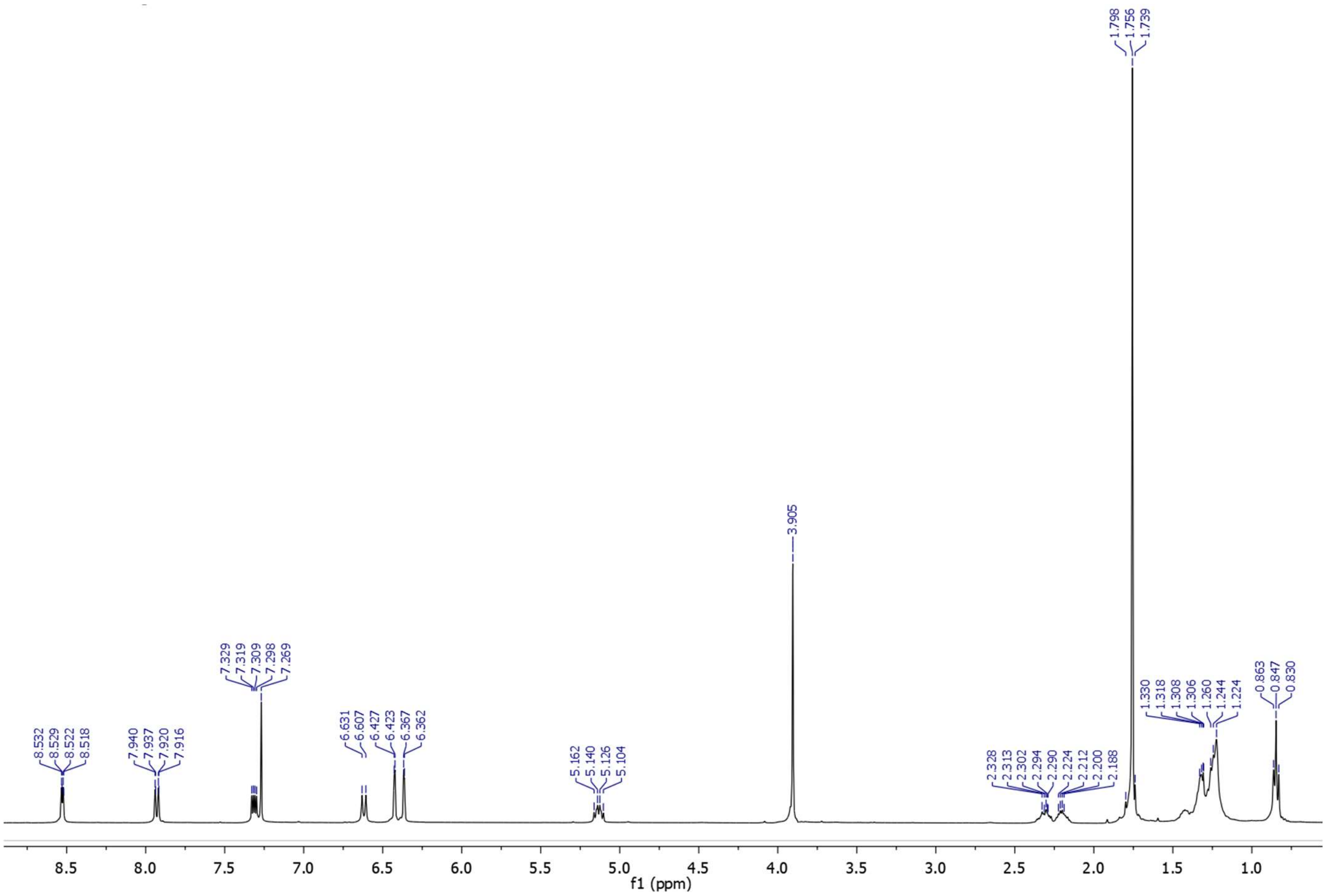

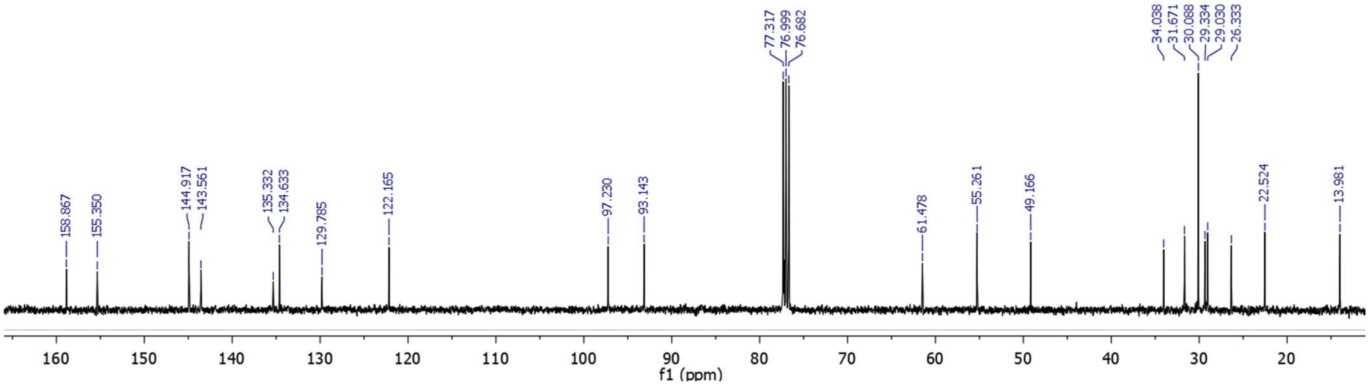

Supplement: Supplementary file 1 [file molecules-26-05530-s001.zip › molecules-1366180-supplementary.pdf]
